# Supplementary material for: Metallopolymer strategy to explore hypoxic active narrow-bandgap photosensitizers for effective cancer photodynamic therapy
Source: Nat Commun. 2024 Jan 2;15:170. doi: 10.1038/s41467-023-43890-z (PMC10762066; doi:10.1038/s41467-023-43890-z)
Supplement: Supplementary file 1 — Supplementary Information [file 41467_2023_43890_MOESM1_ESM.pdf]

# Supplementary Information

## Metallopolymer Strategy to Explore Hypoxic Active Narrow-Bandgap Photosensitizers for Effective Cancer Photodynamic Therapy

Zhao Zhang[1], Zixiang Wei[1], Jintong Guo[1], Jinxiao Lyu[1], Bingzhe Wang[2], Gang Wang[2], Chunfei Wang[1], Liqiang Zhou[1,3], Zhen Yuan[1,3], Guichuan Xing[2], Changfeng Wu[4], Xuanjun Zhang[1,3]*

[1]Cancer Centre and Centre of Reproduction, Development and Aging, Faculty of Health Sciences, University of Macau, Macau SAR, 999078, China.

[2]Institute of Applied Physics and Materials Engineering, University of Macau, Macau SAR 999078, China.

[3]MOE Frontiers Science Centre for Precision Oncology, University of Macau, Macau SAR, 999078, China

[4]Department of Biomedical Engineering, Southern University of Science and Technology, Shenzhen, Guangdong,518055, China

E-mail: xuanjunzhang@um.edu.mo

# Table of Contents

Materials and Measurements ........ 3
Synthesis of Monomers and Polymer ........ 4
Gel Permeation Chromatography ........ 12
Uv/Vis Absorption and Fluorescence Spectra ........ 12
MPdot Fabrication ........ 13
MPdot Size and Surface Potential ........ 13
Electron Paramagnetic Resonance Spectra ........ 14
Cyclic voltammetry ........ 14
ROS Generation in Solution ........ 14
Transient Absorption Spectra ........ 15
Photoacoustic Effect in Solution ........ 16
Cell Culture Conditions ........ 17
*In vitro* ROS Assay ........ 17
Cell Apoptosis Assay ........ 18
Calcein-AM/PI Assay ........ 18
Cell Viability Assay ........ 19
Western Blot Assay ........ 19
MPdot's cRGDyK Peptide Bioconjugation for *In Vivo* Therapy ........ 20
*In Vivo* Photoacoustic Imaging ........ 20
*In vivo* Anti-tumor Therapy ........ 21
$^{1}$HNMR, $^{13}$CNMR, and Mass Spectra ........ 49
Supplementary References ........ 59

## Materials and Measurements

All chemicals and reagents from Sigma–Aldrich and DPP-Sn from Derthon Optoelectronics Materials were used as received from the supplier unless otherwise stated. $^{1}H$ and $^{13}C$ NMR spectra were measured on a Bruker AV-400 MHz NMR spectrometer with chemical shift reported in parts per million (ppm, δ). Mass spectra were recorded on Bruker Microflex MALDI-TOF system. Uv-Vis absorption spectra were measured on a Shimadzu UV-1800 spectrometer. Photoluminescence spectra were conducted on the Horiba Fluorolog-3 spectrofluorometer. Gel permeation chromatography was run on Malvern Viscotek TDA 305. Particle sizes and surface potential measurements were performed on the Malvern Zetasizer Nano ZS. The cell culture serum and medium were purchased from Gibco. The FITC Annexin V/PI Apoptosis Detection Kit was purchased from BD Biosciences. The Calcein-AM/PI Double Stain Kit was purchased from Beyotime. HeLa cells and 4T1 cells were obtained from the Faculty of Health Science, University of Macau. All the cells were incubated in Thermo Fisher Forma Series 3 Water Jacketed $CO_2$ incubator. Confocal laser scanning microscope (CLSM) was performed on Nikon A1R Confocal System. Female BALB/c mice (5 weeks old) were provided by the animal facility of the University of Macau. All animal procedures were approved by the Institutional Animal Care and Use Committee of the University of Macau (approval number: UMARE-013-2022). HpoxyprobeTM-1 Green Kit was purchased from HPI (Hypoxyprobe, Inc, MA, USA). Photoacoustic characterization in solution and imaging *in vivo* were performed on the Vevo LAZR system (FUJIFILM VisualSonics, Toronto, Canada). The femtosecond transient absorption (TA) spectra of different samples were taken using an Ultrafast System HELIOS TA spectrometer.

**Synthesis of Monomers and Polymer**

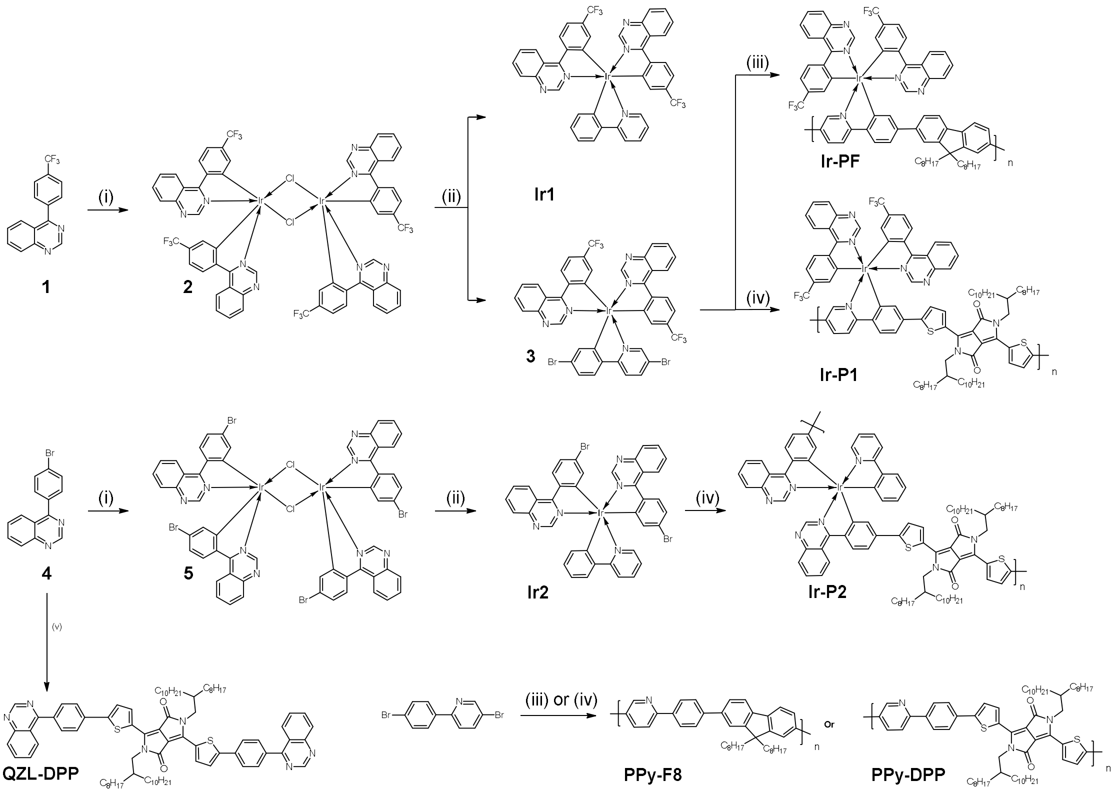


Supplementary Figure 1: Synthesis routine of the monomer and polymer

Reagent and condition:

(i). $IrCl_3$, 2-ethoxyethanol/$H_2O$, 115 °C, 16 h, 86%.

(ii). Silver trifluoromethanesulfonate, 2-ethoxyethanol, 115 °C, 16 h, 19.8% ~ 42%.

(iii). $Pd_2(dba)_3$, P(*o*-tol)$_3$, Toluene, 115 °C, 20 h, 77% ~ 82%.

**Synthesis of Compound 1.**

4-(Trifluoromethyl)phenyl boronic acid (5.77 g, 30.38 mmol, 1eq), 4-chloroquinazoline (5.00 g, 30.38 mmol, 1.00eq), $Pd[PPh_3]_4$ (672.05 mg, 1.82 mmol, 0.06 eq) and $Na_2CO_3$ (9.66 g, 91.13 mmol, 3.00eq) was added into a 250 mL three-neck round bottle equipped with a condenser and a magnetic stirrer. The system was degassed by replacing air with $N_2$ three times before the addition of 120 mL $N_2$-purged blend solution of THF/water (2:1). The mixture was then refluxed at 85 °C for 24 hours. The mixture was extracted with dichloromethane (50 mL twice), dried with anhydrous $MgSO_4$, and concentrated under reduced pressure to afford a yellow crude solid. The product was purified with a silica gel column eluted

with hexane/dichloromethane (1:1) to afford a pale yellow powder (7.66 g, 92%).$^{1}$H NMR (400 MHz, $d_1$-Chloroform) δ ppm: 9.41 (s, 1H), 8.16 (d, J = 8.4 Hz, 1H), 8.04 (d, J = 8.4 Hz, 1H), 7.96 (t, J = 8.0 Hz, 1H), 7.92 (d, J = 8.0 Hz, 2H), 7.85 (d, J = 8.4 Hz, 2H), 7.65 (t, J = 7.6 Hz, 1H).

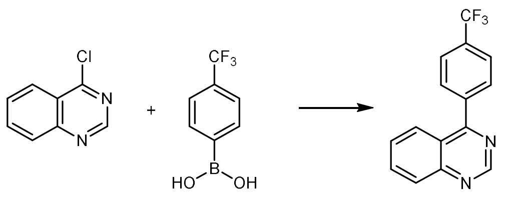


**1**

**Synthesis of Compound 2.**

**Compound 1** (2.00 g, 7.29 mmol, 1eq) and $IrCl_3$ (1.09 g, 3.65 mmol, 0.50 eq) were suspended in 40 mL solution of 2-ethoxyethanol/water (4:1) in a 100 mL three-neck round bottle equipped with a condenser and a magnetic stirrer. The system was degassed by replacing air with $N_2$ three times. The mixture was then refluxed at 115 °C for 16 hours. The mixture was then poured into water (100 mL) and centrifuged and the solid was washed with methanol until the supernatant was colorless. The crude red product could be used without further purification. $^1$H NMR (400 MHz, $d_1$-Chloroform) δ ppm: 9.47 (s, 4H), 8.92 (d, J = 8.4 Hz, 4H), 8.42 (d, J = 8.4 Hz, 4H), 8.16 (m, 8H), 7.98 (t, J = 6.8 Hz, 4H), 7.18 (d, J = 8.4 Hz, 4H), 6.31 (s, 4H).

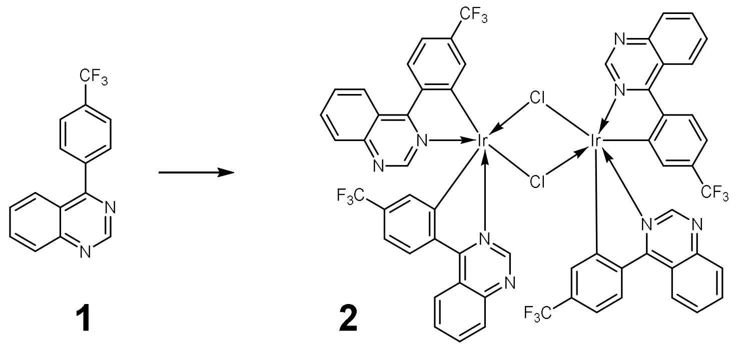


**Synthesis of Compound Ir1.**

**Compound 2** (200.00 mg, 129.18 μmol, 1eq), phenylpyridine (40.10 mg, 258.35 μmol, 2.00 eq), and silver triflate (62.24 mg, 258.35 μmol, 2.00 eq) were suspended in 2 mL $N_2$-purged 2-ethoxyethanol in

a 10 mL tube. The mixture was degassed by replacing the air with $N_2$ three times. The mixture was then refluxed at 115 °C for 16 hours. The mixture was poured into water and extracted with ethyl ether, and then concentrated under reduced pressure and purified with a silica gel column eluted with dichloromethane/methanol (100:1) to afford dark red powder (45.7 mg, 19.8%). $^{1}$H NMR (400 MHz, $d_4$-Acetone) δ ppm: 9.08 (t, J= 11.6 Hz, 2H), 8.87 (s, 1H), 8.78 (dd, J = 13.6 Hz, 2H), 8.47 (s, 1H), 8.28 (d, J = 7.6 Hz, 1H), 8.05 (m, 3H), 7.95 (m, 6H), 7.43 (d, J = 8.4 Hz, 1H), 7.34 (d, J = 8.4 Hz, 1H), 7.15 (t, J = 6.8 Hz, 1H), 7.06 (s, 1H), 7.00 (td, J = 7.2 Hz, 1H), 6.93 (d, J = 7.2 Hz, 1H), 6.90 (s, 1H), 6.65 (d, J = 7.2 Hz, 1H). $^{13}$C NMR (125 MHz, $d_4$-Acetone) δ ppm: 181.34, 177.23, 173.12, 172.18, 168.65, 167.47, 156.39, 152.16, 150.15, 149.20, 146.54, 146.38, 139.44, 138.66, 135.26, 132.43, 131.43, 130.24, 128.33, 126.51, 124.63, 123.01, 119.49, 117.12. Maldi-TOF (m/z): [M]+ calcd. For C41H24F6IrN5, 892.89; found, 892.89.

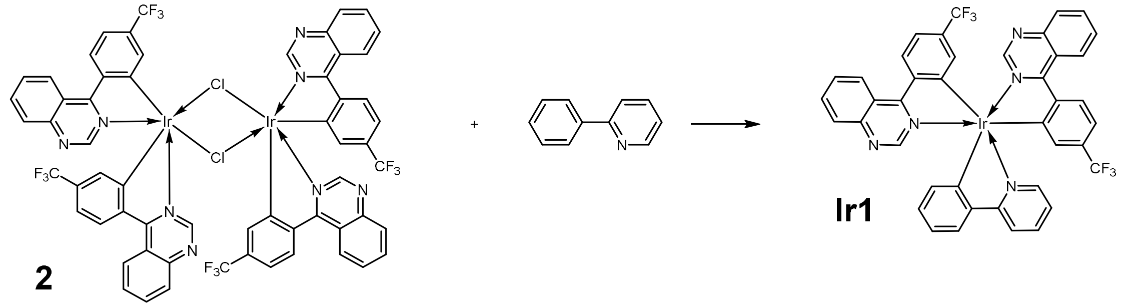


**Synthesis of Compound 3.**

**Compound 3** was synthesized similarly to **Ir1**. **Compound 2** (200.00 mg, 129.18 μmol, 1eq), 5-bromo-2-(4-bromophenyl)-pyridine (80.86 mg, 258.35 μmol, 2.00 eq) and silver triflate (62.24 mg, 258.35 μmol, 2.00 eq) was suspended in 2 mL $N_2$-purged 2-ethoxyethanol in a 10 mL tube. The mixture was degassed by replacing the air with $N_2$ three times. The mixture was then refluxed at 115 °C for 16 hours. The mixture was poured into water and extracted with ethyl ether, and then concentrated under reduced pressure and purified with a silica gel column eluted with dichloromethane/methanol (100:1) to afford dark red powder (114.00 mg, 42.0%). $^{1}$H NMR (400 MHz, $d_4$-Acetone) δ ppm: 9.10 (t, J = 8.4 Hz, 2H), 8.74 (d, J = 8.4 Hz, 1H), 8.68 (d, J = 10.0 Hz, 2H), 8.62 (s, 1H), 8.28 (d, J = 9.2 Hz, 1H), 8.09 (m, 6H), 7.98 (m, 2H), 7.87 (d, J = 8.4 Hz, 1H), 7.46 (d, J = 8.4 Hz, 1H), 7.35 (d, J = 9.6 Hz, 1H), 7.31 (s, 1H),

7.15 (d, J = 6.4 Hz, 1H), 7.13 (s, 1H), 6.78 (s, 1H). $^{13}$C NMR (125 MHz, $d_4$-Acetone) δ ppm: 173.73, 173.27, 167.36, 165.99, 165.04, 161.81, 154.49, 153.82, 152.45, 152.32, 151.25, 149.03, 148.89, 143.88, 142.22, 139.51, 136.36, 136.29, 134.08, 133.88, 135.53, 133.20, 133.12, 132.90, 132.81, 130.68, 130.46, 130.39, 128.19, 127.88, 127.85, 126.73, 125.92, 123.59, 122.54, 119.55, 119.11, 118.92. Maldi-TOF (m/z): [M]+1 calcd. For C41H22Br2F6IrN5, 1050.68; found, 1051.166.

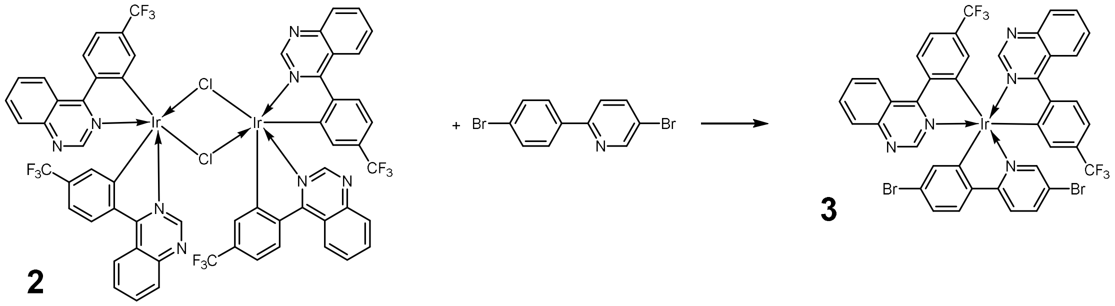


2

3

**Synthesis of Ir-P1**.

**Ir-P1** was synthesized according to the reported literature with minor modifications. **Compound 3** (200 mg, 0.19 mmol, 1eq), DPP-Sn (230.5 mg, 0.194 mmol, 1.02 eq), $Pd_2(dba)_3$ (5.2 mg, 5.70 μmol, 0.03 eq), and P(*o*-tol)$_3$ (7.0 mg, 22.8 mmol, 0.12 eq) was added into a 35 mL Schlenk tube with a magnetic stirrer. The system was degassed by replacing the air with $N_2$ three times before the addition of 5 mL $N_2$-purged dry toluene. The mixture was then refluxed at 115 °C for 60 hours. 2-Bromothiophene (0.1 mL) was added to endcap the polymer under the $N_2$ stream, and the mixture was refluxed for another 12 hours. The mixture was cooled to room temperature and precipitated in the methanol (100 mL) to afford a purple-black fiber. The crude solid was collected and dried before being dissolved with chloroform (10 mL) and precipitated in methanol for the second time. The crude solid was purified with a short $Al_2O_3$ gel column with chloroform as eluent and concentrated to 10 mL. The concentrated mixture was precipitated in the methanol and the purple-black fiber was collected and dried (245.5 mg, 72.4%). Elemental Analysis calculated: C, 65.68%; H, 6.34%; S, 3.76%; N, 5.60%, found: C, 64.87%; H, 5.835%; S, 3.612%; N, 5.41%. Inductively coupled plasma-optical emission spectrometry (ICP-OES) calculated: Ir, 10.57%, found: Ir, 9.380%.

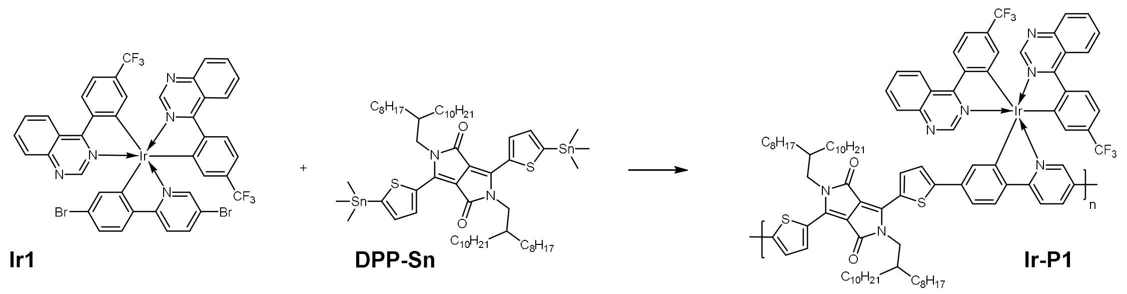


**Synthesis of PPy-DPP**.

**PPy-DPP** was synthesized similarly to **Ir-P1**. 5-bromo-2-(4-bromophenyl)-pyridine (80 mg, 255.60 μmol, 1eq), DPP-Sn (284.6 mg, 255.60 μmol, 1.0 eq), $Pd_2(dba)_3$ (7.0 mg, 7.67 μmol, 0.03 eq) and P(*o*-tol)$_3$ (9.3 mg, 30.67 μmol, 0.12 eq) was added into a 35 mL Schlenk tube with a magnetic stirrer. The system was degassed by replacing the air with $N_2$ three times before the addition of 5 mL $N_2$-purged dry toluene. The mixture was then refluxed at 115 °C for 60 hours. 2-Bromothiophene (0.1 mL) was added to endcap the polymer under the $N_2$ stream, and the mixture was refluxed for another 12 hours. The mixture was cooled to room temperature and precipitated in the methanol (100 mL) to afford a purple-black fiber. The crude solid was collected and dried before being dissolved with chloroform (10 mL) and precipitated in methanol for the second time. The crude solid was collected and dried (201.5 mg, 75.6%).

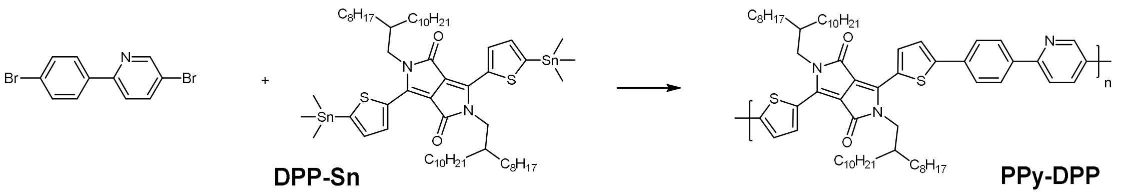


**Synthesis of Compound 4.**

4-bromophenyl boronic acid (3.66 g, 18.23 mmol, 1eq), 4-chloroquinazoline (3.00 g, 18.23 mmol, 1.00eq), Pd[$PPh_3$]$_4$ (201.61 mg, 0.546 mmol, 0.03 eq) and $Na_2CO_3$ (5.80 g, 54.68 mmol, 3.00eq) was added into a 250 mL three-neck round bottle equipped with a condenser and a magnetic stirrer. The system was degassed by replacing air with $N_2$ three times before the addition of 120 mL $N_2$-purged blend solution of THF/water (2:1). The mixture was then refluxed at 85 °C for 24 hours. The mixture was extracted with dichloromethane (50 mL twice), dried with anhydrous $MgSO_4$, and concentrated under reduced pressure to afford a yellow crude solid. The product was purified with a silica gel column eluted with hexane/dichloromethane (2:3) to afford a pale-yellow powder (4.42 g, 85%).$^1$H NMR (400 MHz,

$d_6$-Acetone) δ ppm: 9.36 (s, 1H), 8.20 (d, J = 8.4 Hz, 1H), 8.14 (d, J = 8.4 Hz, 1H), 8.08 (t, J = 6.8 Hz, 1H), 7.86(m, 4H), 7.79 (t, J = 6.8 Hz, 1H).

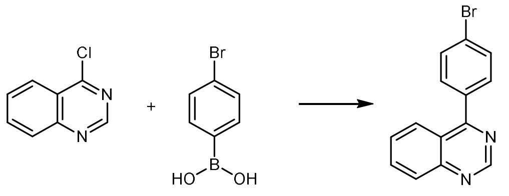


4

**Synthesis of Compound 5.**

**Compound 4** (2.00 g, 7.01 mmol, 1eq) and $IrCl_3$ (1.05 g, 3.51 mmol, 0.50 eq) were suspended in 40 mL solution of 2-ethoxyethanol/water (4:1) in a 100 mL three-neck round bottle equipped with a condenser and a magnetic stirrer. The system was degassed by replacing air with $N_2$ three times. The mixture was then refluxed at 115 °C for 16 hours. The mixture was then poured into water (100 mL) and centrifuged and the solid was washed with methanol until the supernatant was colorless. The crude red product could be used without further purification. $^1$HNMR spectra failed to acquire due to the extremely poor solubility in the commonly used solvent.

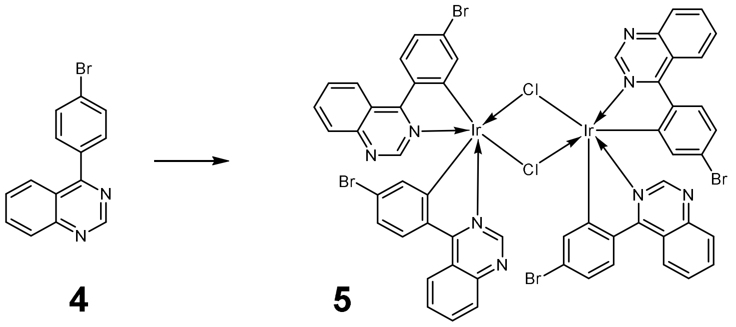


4 5

**Synthesis of Compound Ir2.**

**Ir2** was synthesized similarly to **Ir1**. **Compound 5** (500.00 mg, 314.09 μmol, 1eq), 5-bromo-2-(4-bromophenyl)-pyridine (97.49 mg, 628.19 μmol, 2.00 eq) and silver triflate (161.40 mg, 628.19 μmol, 2.00 eq) was suspended in 2 mL $N_2$-purged 2-ethoxyethanol in a 10 mL tube. The mixture was degassed by replacing the air with $N_2$ three times. The mixture was then refluxed at 115 °C for 16 hours. The mixture was poured into water and extracted with ethyl ether, and then concentrated under reduced

pressure and purified with a silica gel column eluted with dichloromethane/methanol (100:1) to afford dark red powder (298.78 mg, 52.0%). $^{1}$H NMR (400 MHz, d$_6$-Acetone) δ ppm: 9.08 (d, J = 8.4 Hz, 1H), 9.04 (d, J = 8.8 Hz, 1H), 8.80 (s, 1H), 8.61 (d, J = 8.8 Hz, 1H), 8.57 (d, J = 8.4 Hz, 1H), 8.39 (s, 1H), 8.30 (d, J = 8.4 Hz, 1H), 8.05 (m, 3H), 8.01 (m, 3H), 7.94 (m, 3H), 7.38 (dd, J = 8.8 Hz, 1H), 7.31 (dd, J = 8.8 Hz, 1H), 7.22 (t, J = 7.2 Hz, 1H), 7.03 (t, J = 7.6 Hz, 1H), 6.98 (t, J = 7.2 Hz, 1H), 6.95 (d, J = 2.0 Hz, 1H), 6.84 (d, J = 2.0 Hz, 1H), 6.79 (d, J = 6.8 Hz, 1H). $^{13}$C NMR (125 MHz, d$_1$-Chloroform) δ ppm: 183.47, 176.67, 171.42, 168.851, 168.124, 152.86, 151.14, 149.87, 149.00, 138.34, 134.43, 133.48, 129.82, 128.36, 126.22, 124.83, 122.59, 119.70. Maldi-TOF (m/z): [M]+1 calcd. For C39H24Br2IrN5, 913.00; found, 8914.909.

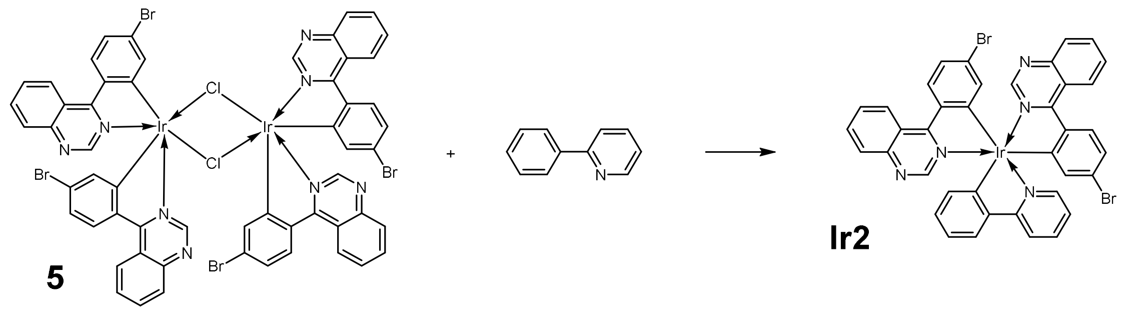


**Synthesis of Ir-P2**.

**Ir-P2** was synthesized similarly to **Ir-P1**. **Compound 4** (80 mg, 0.87 mmol, 1eq), DPP-Sn (105.82 mg, 0.87 mmol, 1.02 eq), $Pd_2(dba)_3$ (5.2 mg, 5.70 μmol, 0.03 eq), and P(*o*-tol)$_3$ (7.0 mg, 22.8 mmol, 0.12 eq) was added into a 35 mL Schlenk tube with a magnetic stirrer. The system was degassed by replacing the air with $N_2$ three times before the addition of 5 mL $N_2$-purged dry toluene. The mixture was then refluxed at 115 °C for 60 hours. 2-Bromothiophene (0.1 mL) was added to endcap the polymer under the $N_2$ stream, and the mixture was refluxed for another 12 hours. The mixture was cooled to room temperature and precipitated in the methanol (100 mL) to afford a purple-black fiber. The crude solid was collected and dried before being dissolved with chloroform (10 mL) and precipitated in methanol for the second time. The crude solid was purified with a short $Al_2O_3$ gel column with chloroform as eluent and concentrated to 10 mL. The concentrated mixture was precipitated in the methanol and the purple-black fiber was collected and dried (117.6 mg, 83.2%). Elemental Analysis calculated: C, 69.94%; H, 7.09%;

S, 4.17%; N, 5.80%; found: C, 69.75%; H, 6,823%; S, 3.78%; N, 5.29%; ICP-OES calculated: Ir, 10.93%; found: Ir, 9.7852%.

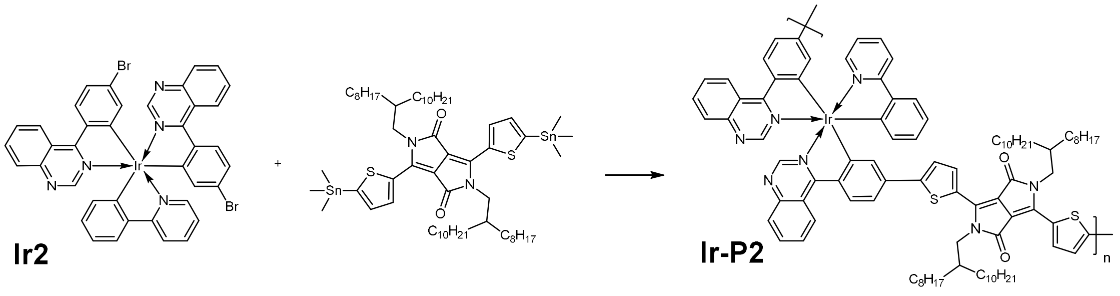


**Synthesis of Ir-PF8**, **Ir-PTBT**, **Ir-P44** and **Ir-P37**.

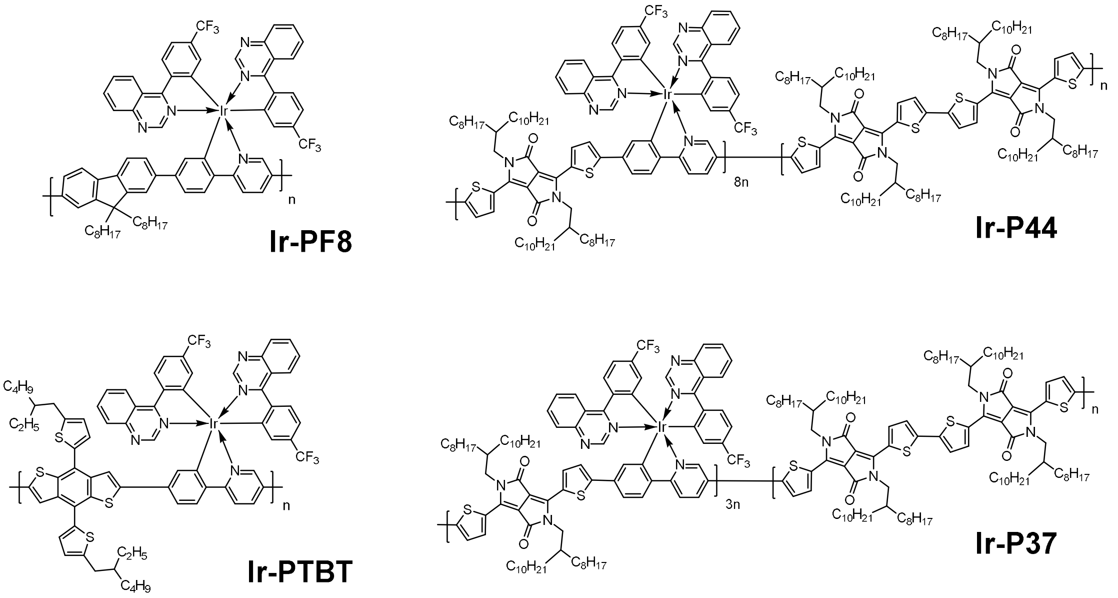


**Ir-PF8**, **Ir-PTBT**, **Ir-P44** and **Ir-P37** were synthesized similarly to **Ir-P1**.

**Synthesis of QZL-DPP**.

**QZL-DPP** was synthesized similarly to **Ir-P1**. **Compound 4** (25.0 mg, 87.67 μmol, 1eq), DPP-Sn (50.0 mg, 87.67 μmol, 0.48 eq), $Pd_2(dba)_3$ (2.41 mg, 2.63 μmol, 0.03 eq) and P(*o*-tol)$_3$ (3.20 mg, 10.52 μmol, 0.12 eq) was added into a 10 mL Schlenk tube with a magnetic stirrer. The system was degassed by replacing the air with $N_2$ three times before the addition of 3 mL $N_2$-purged dry toluene. The mixture was then refluxed at 115 °C for 18 hours. The mixture was cooled to room temperature and purified with a silica gel column eluted with dichloromethane/methanol (100:10) to afford dark powder (85.7 mg, 76.97%). $^1$H NMR (400 MHz, $d_8$-THF) δ ppm: 9.30 (s, 2H), 9.20 (d, 2H), 8.23 (d, 2H), 8.09 (d, 2H), 7.97

(m, 10H), 7.79 (d, 2H), 7.67 (m, 2H), 4.16 (d, 4H), 1.37 (m, 68H), 0.84 (m, 12H). $^{13}C$ NMR (125 MHz, $d_8$-THF) δ ppm: 167.81, 162.22, 155.79, 152.65, 149.35, 132.21, 128.73, 127.64, 126.86, 126.45, 123.96, 109.55, 46.99, 39.18, 33.05, 30.52, 23.72, 14.63, 14.61.

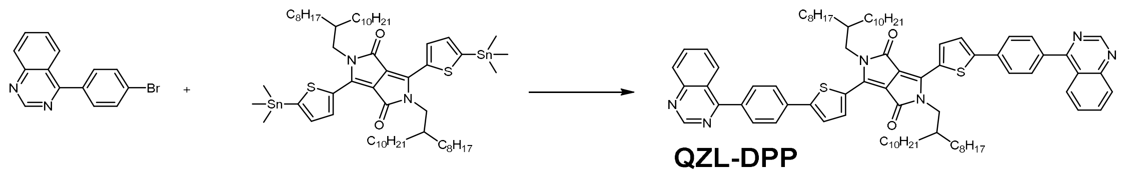


**Gel Permeation Chromatography**

A 100 μL aliquot of the polymer solution, which had a concentration of 1 mg/mL in tetrahydrofuran (THF), was processed through the Malvern Viscotek TDA 305 system at a flow rate of 1 mL/min. The data gathered was subsequently analyzed using the Malvern Viscotek software and calibrated with a standard curve. This standard curve was derived from the testing of standard polystyrenes with diverse molecular weights (170,000, 103,500, 77,000, 29,600, 13,400, 6,040, 2,560, and 1,200, respectively).

**Uv/Vis Absorption and Fluorescence Spectra**

The compounds **Ir1**, **Ir2**, **DPP**, and **QZL-DPP** were dissolved in dichloromethane (DCM) to form a 1 mM solution, which was subsequently diluted to a concentration of 2.85 μM. Similarly, **Ir-P1**, **PPy-DPP**, and **Ir-P2** were dissolved in DCM to make a 1mg/mL solution. For **Ir-P1**, this metallopolymer solution was then diluted to achieve concentrations of 5.00 μg/mL (equivalent to 2.80 μM in one repeating unit), 2.92 μg/mL for **PPy-DPP**, and 4.54 μg/mL for **Ir-P2**. The UV/Vis spectra were obtained by measuring 2 mL of each of these solutions. For the fluorescence spectra, the solutions were additionally diluted by a factor of 5 before measurement.

**MPdot Fabrication**

MPdot was fabricated by the precipitation method. **Ir-P1**, **Ir-P2**, **Ir-P44**, **Ir-P37**, **Ir-PF8**, **Ir-PTBT**, **PPy-DPP**, **PPyF8**, **PPy-TBT** and PSMA (Sigma-Aldrich, 442402-250G-A, #08728HN) were dissolved with THF at a concentration of 1 mg/mL, respectively, and stored at -20 °C for further use. 250μL **Ir-P1** solution and 100 μL PSMA solution were added into a glass bottle and diluted with THF to 5 mL and injected into 25 mL of deionized water under ultrasonication and sonicated for 10 mins to afford a homogenous dark green solution. The solution was dialyzed with deionized water (2L x 6 times) for 48 hours with a cut-off of 100,000 Da. After dialysis, the solution was filtered with a 0.22 μm filter and concentrated under reduced pressure to afford a dark green solution. The concentration of the solution is based on the metallopolymer and calibrated with linear-fitted Uv/Vis concentration-dependent absorption spectra. MPdot solution can be stored at 4 °C for further use.

The blue fluorescence emitting MPdot was fabricated similarly, with a blend solution of 250 μL **Ir-P**: 50 μL **PPyF8**: 100 μL PSMA.

**PPyF8**

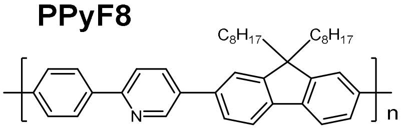


Other polymeric nanoparticles were fabricated in a similar procedure.

**MPdot Size and Surface Potential**

MPdot solution was diluted to 50 μg/mL and 2 mL was measured on dynamic light scattering (Malven Zetasizer Nano ZS).

**Electron Paramagnetic Resonance Spectra**

The EPR measurements were conducted using a Bruker Model A300 at ambient temperature. All samples were prepared under identical conditions, with PSs (0.5 mM) and BMPO or TEMP (100 mM) in a total volume of 200 μL. Different light sources were employed for different compounds: a white light source (50 $mW\cdot cm^{-2}$) for **Ir1**, **Ir2**, **MPdotF8**, and **MPdotTBT**; a 660 nm laser (800 $mW\cdot cm^{-2}$) for **MPdot1** and **MPdot2**; and an 808 nm laser (1 $W\cdot cm^{-2}$) for **MPdot44** and **MPdot37**. Before the EPR spectra measurements, the samples were exposed to light for 5 minutes. For time-dependent spectra, the samples were irradiated, and the spectra were recorded at each time interval. Control groups were prepared in the same manner but without any light exposure.

**Cyclic voltammetry**

The cyclic voltammetry measurements were conducted on a HY 1550A mini electrochemical analyzer. All samples (2 $mg\cdot mL^{-1}$) were evaluated in anhydrous dichloromethane at a scan rate of 100 $mV\cdot s^{-1}$. $Bu_4NPF_6$ was used as an electrolyte at a concentration of 0.2 M. Saturated calomel electrode was used as a reference electrode, with a glassy-carbon electrode used as a working electrode, and Pt wire as a counter electrode; and $Fc/Fc^+$ was used as an external reference. The redox potential level for the standard hydrogen electrode was calculated with the equation: $E_{NHE} = E_{SCE} + 0.24$ V.

**ROS Generation in Solution**

The tetrahydrofuran solution of DBPF (10 mM), Ce6 (1 mM), **Ir1** (1 mM), **Ir-P1** (1 mg/mL), **DPP-PPy** (1 mg/mL), **Ir2** (1 mM), **Ir-P2** (1 mg/mL), and **QZL-DPP** (1 mM) were prepared as stock solution.

MPdot (1 mg/mL) in water was prepared. The $H_2O$ solutions of scavengers, Tiron (1 M) and *t*-butanol (1 M), were prepared for use.

The general procedure for ROS detection in DCM:

The tetrahydrofuran solution of PS was diluted in DCM to a final concentration of 2.80 μM and the Uv/Vis absorption spectra were recorded. 10 μL DPBF solution was added to the solution (final concentration was 50 μM) with its absorption spectra recorded. The mixture solution was irradiated with a 680 nm laser (5 $mW \cdot cm^{-2}$) and its absorption spectra were recorded every 30 s. A time-dependent absorbance curve at 410 nm was drawn. The ROS quantum yield was calculated according to the following equation[61]:

$$\emptyset_{complex} = \emptyset_{Ce6} * \left(\frac{K_{complex}}{K_{Ce6}}\right) * \left(\frac{F_{Ce6}}{F_{complex}}\right) \text{ ---- } (1)$$

where $K$ is the decomposition rate constants of DPBF calculated from the absorption decrease at 410 nm, in the presence of various PSs; $F = 1\text{–}10^{-OD}$, and OD is the absorbance at 680 nm for individual PS.

The general procedure for ROS scavenging in tetrahydrofuran/water (1:1, v/v):

MPdot was diluted in tetrahydrofuran/water (1:1) to 5.00 μg/mL (2.80 μM, calculated with one repeating unit), and the Uv/Vis absorption spectra were recorded. 10 μL DPBF and 10 μL scavenger solution were added to the mixture solution (final concentration was 50 μM for DPBF and 5 mM for scavenger) with its absorption spectra recorded. The mixture solution was then irradiated with a 680 nm laser (20 $mW \cdot cm^{-2}$ )and its absorption spectra were recorded every 30 s. A time-dependent absorbance curve at 410 nm was drawn.

**Transient Absorption Spectra**

The Ultrafast System HELIOS TA spectrometer was used to capture the femtosecond transient absorption (TA) spectra of various samples. The laser source was provided by the Coherent Astrella-1K-F Ultrafast Ti: Sapphire Amplifier (800nm, 1 kHz, <100 fs). Broadband probe pulses ranging from 450 to 775 nm were created by focusing a small fraction of the fundamental 800 nm laser pulses onto an $Al_2O_3$ plate. The 400-nm pump pulses were produced by doubling the fundamental 800-nm pulses using a BBO crystal.

In the time-profile fittings, we used following formula:

$$y = A + B_1 \exp\left(\frac{-t}{\tau_1}\right) + B_2 \exp\left(\frac{-t}{\tau_2}\right) + B_3 \exp\left(\frac{-t}{\tau_3}\right) \quad \text{----} \quad (2)$$

where B are relative amplitudes, and $\tau_1$, $\tau_2$ and $\tau_3$ represent the lifetimes of different photophysical pathways. In our case, the charge recombination is the last process and derived as $\tau_3$.

**Photoacoustic Effect in Solution**

The photoacoustic imaging in solution was detected on the VEVO LAZR-X system (FUJIFILM VisualSonics, Toronto, Canada). For photoacoustic spectra, the photoacoustic signal was acquired at 680, 685, 700, …, 745, 750 nm (100 ppm **MPdot1c** or **MPdot2c** in PBS, PA-Model(single), 100% power, 40-dB gain, 40-MHz frequency). For concentration-dependent photoacoustic spectra, the photoacoustic signal was acquired at 0, 6.25, 12.5, 25, and 50 ppm of **MPdot1c** or **MPdot2c** in PBS, (680 nm, PA-Model(single), 100% power, 40-dB gain, 40-MHz frequency). The intensity of the photoacoustic signal was determined by delineating the region of interest by the imaging system.

**Cell Culture Conditions**

HeLa cells were incubated under 5.0% $CO_2$ and 21.0% $O_2$ at 37 °C in a humidified atmosphere in Dulbecco's Modified Eagle Medium (DMEM, Gibco BRL) with 10% (v/v) fetal bovine serum (BFS, Gibco BRL) and 100 µg/mL streptomycin and penicillin (Gibco BRL). The 4T1 cells were incubated under 5.0% $CO_2$ and 21.0% $O_2$ at 37 °C in a humidified atmosphere in RPMI 1640 Medium (Gibco BRL) with 10% (v/v) fetal bovine serum (BFS, Gibco BRL) and 100 µg/mL streptomycin and penicillin (Gibco BRL).

For hypoxic conditions, the $O_2$ level of the incubator was set to 1% with other conditions unchanged.

***In vitro* ROS Assay**

50,000 HeLa cells were seeded in the 3.5 mL confocal microscopy dishes 16 hours in advance and divided into four groups with different treatments. MPdot coated with R8 in deionized water was diluted with DMEM to 20 µg/mL and used to incubate the cells in MPdot groups for 20 hours. All cells were incubated with DHE or DHR 123 in DMEM without FBS for 0.5 hours. The cells were further washed with PBS three times and the laser groups were irradiated with a 680 nm laser (40 $mW \cdot cm^{-2}$) for 5 mins. For the $H_2$DCFDA assay, the MPdot concentration was increased to 60 µg/mL and probe incubation to 1 hour, and irradiation time to 10 min. Then the PBS was replaced with fresh PBS and confocal fluorescence images were taken soon after the laser irradiation using Nikon A1R ($\lambda_{ex}$ =488 nm, $\lambda_{em}$ =515-535 nm).

For ROS assay by using flow cytometry, 50,000 HeLa cells were seeded in 12-well plates 16 hours in advance and divided into four groups with different treatments. MPdot coated with R8 in deionized water was diluted with DMEM to 20 μg/mL and used to incubate the cells in MPdot groups for 20 hours. All cells were then washed with PBS three times and incubated with DHE or DHR 123 (5μM) in DMEM without FBS for 0.5 hours. The cells were further washed with PBS three times and the cells in laser groups were irradiated with a 680 nm laser (40 $mW \cdot cm^{-2}$) for 5 mins. Then all cells were washed with fresh PBS, digested with trypsin, and collected for further detection and analysis using a flow cytometer. For the $H_2DCFDA$ assay, the MPdot concentration was increased to 60 μg/mL and probe incubation to 1 hour, and irradiation time to 10 min. For the scavenging experiments, 1,4-benzoquinone (1 mM) was added with the DHR 123 containing DMEM with other conditions unchanged.

**Cell Apoptosis Assay**

50,000 HeLa cells were prepared in 12-well plates 16 hours in advance and were randomly categorized into four groups for distinct treatments. The cells in the MPdot groups underwent treatment with MPdot (20 μg/mL) for 20 hours. Subsequently, the cells in the laser groups were subjected to a 680 nm laser (100 $mW \cdot cm^{-2}$)for 5 minutes. Following this, all cells were cultivated in fresh medium for an additional 8 hours, then collected and stained with Annexin V-FITC (5 μL for 15 min) and PI (5 μL for 5 min). Finally, the cells population was quantified using flow cytometry with PE and FICT channel analyzed.

**Calcein-AM/PI Assay**

5,000 HeLa cells were prepared in 96-well plates 12 hours in advance. The cells were subsequently treated with MPdot at various concentrations of 0 μg/mL, 4 μg/mL, 8 μg/mL, and 12 μg/mL for 20 hours. Following this, a 680 nm laser (100 mW·cm$^{-2}$) was used to irradiate cells in laser groups for 20 minutes. The cells were then cultivated for an additional 24 hours in fresh DMEM. Subsequently, the cells were treated with a fresh medium containing Calcein-AM (10 μg/mL) and PI (5 μg/mL) for 20 minutes. Finally, the cells were gently rinsed with PBS for CLSM observation.

**Cell Viability Assay**

5,000 HeLa cells were prepared in 96-well plates 12 hours in advance. The cells were then treated with MPdot at various concentrations ranging from 0 μg/mL to 12 μg/mL for 20 hours. The cells in the laser groups were exposed to a 680 nm laser (100 mW·cm$^{-2}$) for 20 minutes, while the cells in the dark group remained untreated. The medium for all groups was subsequently replaced with fresh, complete DMEM, and the cells were allowed to proliferate for an additional 24 hours. This was succeeded by a replacement of the medium with a complete WST-8 containing DMEM medium (10%, v/v, Cell Counting Kit-8, C0039, Beyotime Biotechnology). Following 3 hours, Perkin Victor X5 microplate reader was used to measure the 450 nm absorbance of each well. Excel and GraphPad software (version 9.0.0) were used to determine the viability of the cells in each well and the IC50 value via nonlinear fitting.

**Western Blot Assay**

Protein samples from HeLa cells that underwent different treatments (~ 10 μL) were subjected to polyacrylamide gel electrophoresis (SDS-PAGE, 10%) and subsequently transferred to nitrocellulose membranes. Bovine serum albumin (BSA) was used to block the membranes in blocking buffer

containing a 0.05% Tween, and this was incubated for 1.5 h at room temperature. Primary labeling was conducted by incubating the sample at 4 °C for 12 hours with monoclonal antibody BcL-2 (Lot#B2117, sc-7382, Santa Cruz Biotechnology, Inc) at a 1:2000 dilution, using a blocking buffer. Rabbit anti-Mouse IgG Secondary Antibody (Catalog # **61-6520, Invitrogen**) was used at a 1:1000 dilution as a secondary marker. Reactive protein bands were detected by exposing the membranes to a luminol-hydrogen peroxide solution. Images were captured and analyzed with Image Bio-Rad Lab™ software.

**MPdot's cRGDyK Peptide Bioconjugation for *In Vivo* Therapy**

To a rapidly stirred MPdot solution (2.8 mL, 1.54 mg/mL, with 40% PSMA), added fresh aqueous solution of N-(3-Dimethylaminopropyl)-N'-ethylcarbodiimide hydrochloride (12.4 µL, 7.2 mg/mL) and N-hydroxysuccinimide (4.97 µL, 10.8 mg/mL). The mixture was stirred at room temperature for 2 hours, and the aqueous solution of cRGDyK (28.9 µL, 10 mg/mL, sequence: Cyclo(Arg-Gly-Asp-D-Tyr-Lys from Shanghai RoyoBiotech Co., Ltd) was added. The mixture was further stirred at room temperature for 24 hours before purifying with a Sephadex® G-25 column to remove the free small molecules.

***In Vivo* Photoacoustic Imaging**

Mice bearing 4T1 tumor were injected through the tail vein with cRGDyK-modified **MPdot1c** or **MPdot2c**, and unmodified **MPdot1** or **MPdot2** (400 pp, 100 µL, 2 mg $kg^{-1}$). For time-dependent MPdot accumulation at tumor photoacoustic imaging, the Single model of the Vevo LAZR was used. 0, 3, 7, 12, and 24 hours after MPdot administration, the photoacoustic signal was acquired (680 nm, 100% power,

40-dB gain, 40-MHz frequency). The intensity of the photoacoustic signal in the tumor region was determined by delineating the region of interest by the imaging system.

The Oxy-Hemo imaging mode (40-dB gain, 10.00 mm depth, 12.08 mm width) was used to map the $O_2$ saturation(sO2), in which the 750 nm and 850 nm laser source switched. sO2 and HbT in the epidermal area and tumor area were measured using an imaging system to outline the area of interest.

***In vivo* Anti-tumor Therapy**

4T1 tumor model were performed by subcutaneous injection of $5 \times 10^5$ 4T1 cells in 0.1 mL of PBS into the back of the mice. Seven days after model building, mice were randomly divided into four groups for different treatment. In **MPdotc** groups, a 100 μL solution of cRGDyK-modified **MPdotc** (**MPdot1c** or **MPdot2c**, respectively) at a concentration of 400 μg/mL was injected intravenously via tail (**MPdotc** dose: 2 mg $kg^{-1}$). The remaining mice were injected with 100 μL PBS solution instead. 12 hours later, a 680 nm laser (300 $mW \cdot cm^{-2}$) was used to irradiate the tumor site for 10 minutes.

The mice in the laser-negative group were not treated. The same treatment was repeated on the $12^{th}$ day.

The tumor sizes were measured with calipers calculated using the following formula:

$$V=\frac{\pi}{6}*A*B^2 \text{ ---- } (3)$$

Where A is the long diameter of the tumor and B is the shot diameter.

On the $17^{th}$ day, all the mice were sacrificed with tumors and organs dissected.

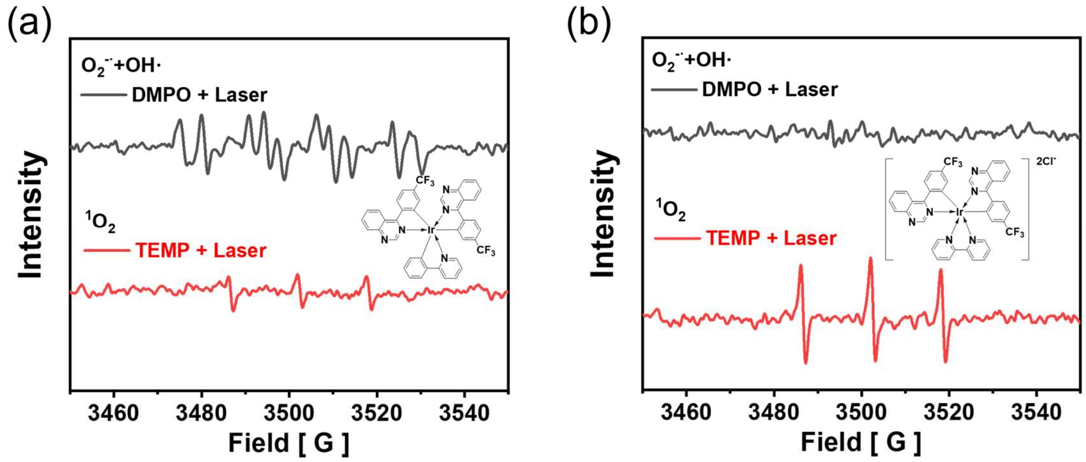


Supplementary Figure 2. The EPR data was obtained from an aqueous solution containing DMPO or TEMP. Experimental conditions: [**Ir1**] = [**Ir-N^N**] = 0.5 mM, [DMPO] = [TEMP] = 100 mM; irradiation for 5 min: white light (50 mW·cm$^{-2}$). (a). **Ir1**; (b). **IrN^N**.

Supplementary Table 1. Polymer weight and Ir content fraction in the polymer.

| Polymer | Mn (Đ) | Mw (Đ) | PDI | PD | Ir (*w*%, found[(a)]/calculated)[(b)] |
|---|---|---|---|---|---|
| **Ir-P1** | 26,698 | 68,149 | 2.55 | 15.0 | 9.38/10.57 |
| **Ir-P2** | 10,920 | 19,207 | 1.76 | 6.75 | 9.79/10.77 |

The polymer weight and polydispersity index (PDI) data are acquired on a gel permeation chromatography (GPC) system. The degree of polymerization (PD) is calculated based on number-average molecular weight and repeating unit. The Ir content fraction data is acquired on an inductively coupled plasma optical emission spectroscopy (ICP-OES) system. (a). measured content fraction; (b). calculated content fraction based on the degree of polymerization.

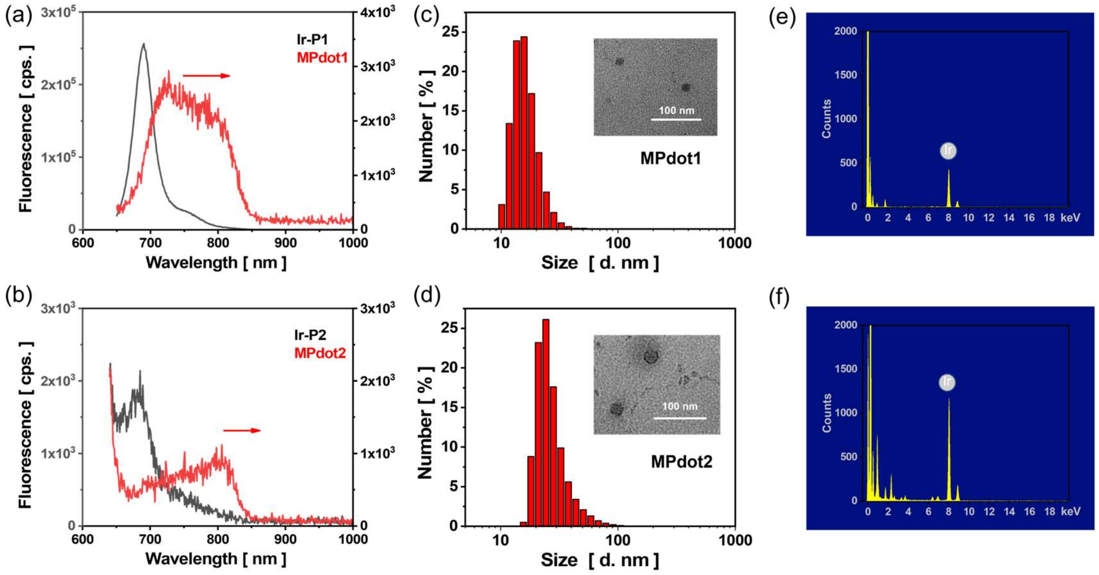


Supplementary Figure 3. Fluorescence spectra of metallopolymers in dichloromethane (black line) and their corresponding MPdot in water (red line). Experimental condition: [**Ir-P1**] = [**MPdot1**] = [**Ir-P2**] = [**MPdot2**] = 0.57 μM. (a). **Ir-P1** and **MPdot1**; (b) **Ir-P2** and **MPdot2**. Particle sizes of **MPdot1** (c) and **MPdot2** (d). Insets in (c) and (d) are TEM images (scale bar: 100 nm). The energy-dispersive X-ray spectroscopy (EDS) of (e). **MPdot1** and (f). **MPdot2**.

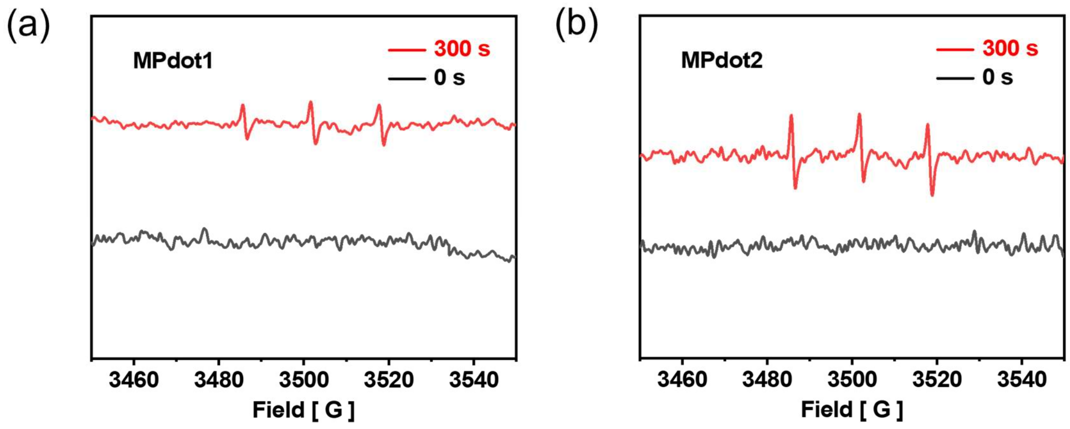


Supplementary Figure 4. EPR data was obtained from an aqueous solution containing TEMP. (a). **MPdot1**; (b). **MPdot2**; irradiation of 680 nm laser (800 mW·cm$^{-2}$) for 5 mins. Experimental conditions: [**MPdot1**] =[**MPdot2**] = 0.5 mM, [TEMP] = 100 mM.

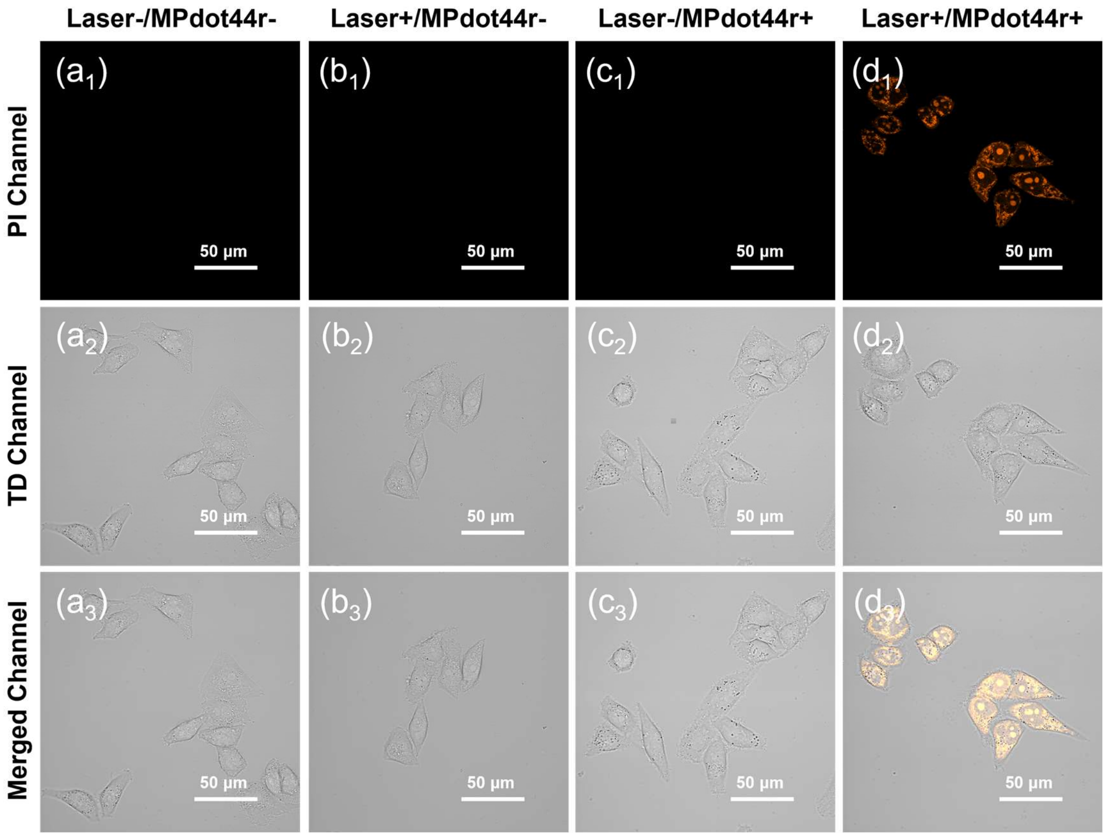


Supplementary Figure 5. $O_2^{\bullet-}$ detection with DHE. Experimental conditions: [**MPdot44r**] = 50 μg/mL with preincubation for 20 hours, [DHE] = 5 μM with incubation for 30 mins, 808 nm laser irradiation (1 W·cm$^{-2}$) for 5 mins. All experiments were repeated three times independently with similar results.

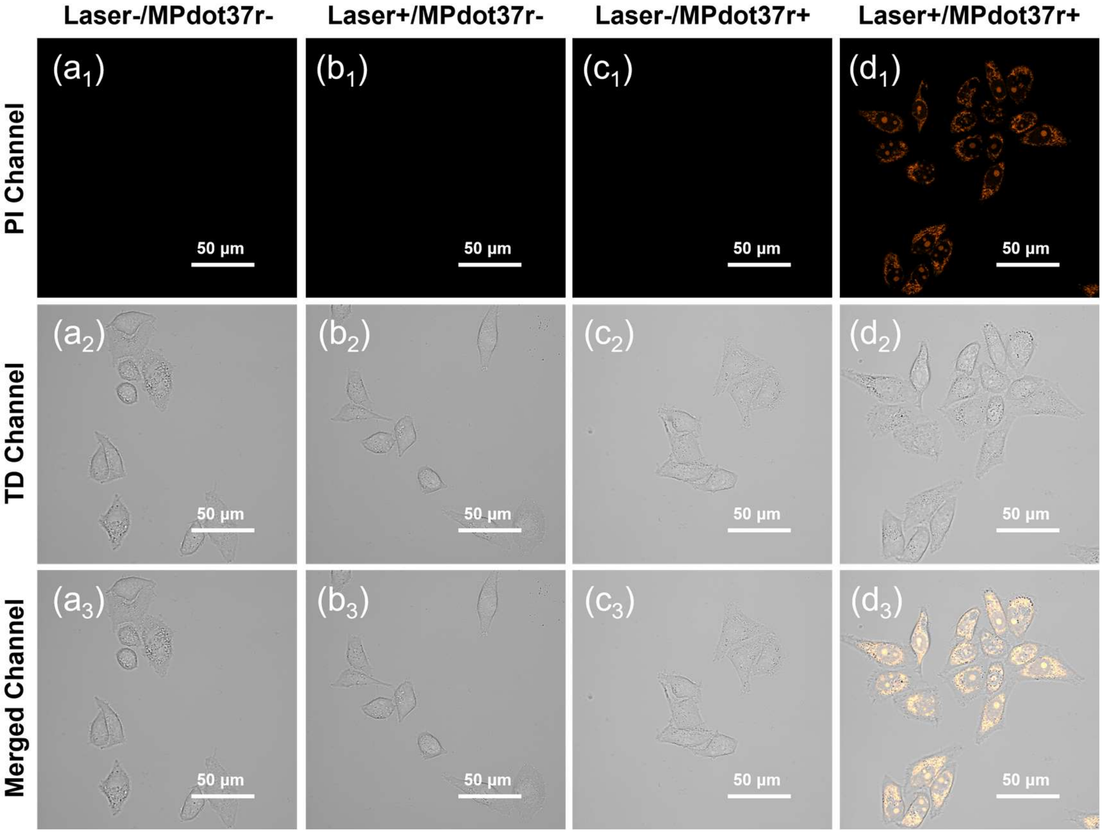


Supplementary Figure 6. $O_2^{\bullet-}$ detection with DHE. Experimental conditions: [**MPdot37r**] = 50 μg/mL with preincubation for 20 hours, [DHE] = 5 μM with incubation for 30 mins, 808 nm laser irradiation (1 W·cm$^{-2}$) for 5 mins. All experiments were repeated three times independently with similar results.

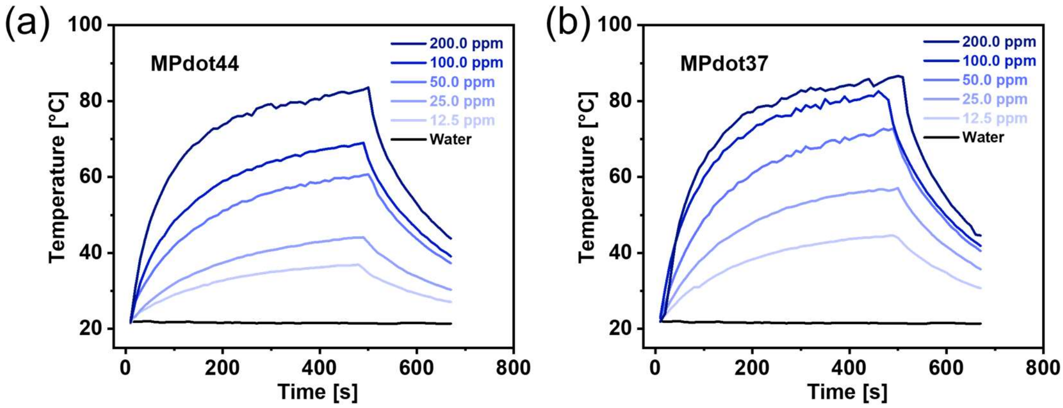


Supplementary Figure 7. Concentration-dependent photothermal effects of **MPdot44** (a) and **MPdot37** (b). Experimental condition: 808 nm laser, 1 W·cm$^{-2}$, 250 μL in 96-well plate, and data recorded every 10 s.

Supplementary Table 2. The absorption and ROS generation properties of PSs

| PS | $\varepsilon_{max}/10^4\ M^{-1}cm^{-1}$ | $K_{PS}/K_{Ce6,\ 473\ nm}$ [(a)] | $K_{PS}/K_{Ce6,\ 680\ nm}$ [(b)] |
|---|---|---|---|
| **Ir1** | 3.68 (302 nm) | 0.018 | - |
| **PPy-DPP** | 16.21 (719 nm) | 0.150 | 0.019 |
| **Ir-P1** | 11.93 (669 nm) | 24.626 | 1.609 |
| **Ir2** | 2.46 (306 nm) | 1.028 | - |
| **DPP** | 2.54 (549 nm) | 2.940 | - |
| **Ir-P2** | 9.00 (680 nm) | 12.328 | 1.593 |
| **QZL-DPP** | 5.93 (610 nm) | 0.370 | - |
| **Ce6** | 2.57 (680 nm) | 1 | 1 |

The reaction rates are calculated from DPBF's absorption at 411 nm and Ce6's reaction rate is set to 1.

(a). 473 nm laser. (b). 680 nm laser.

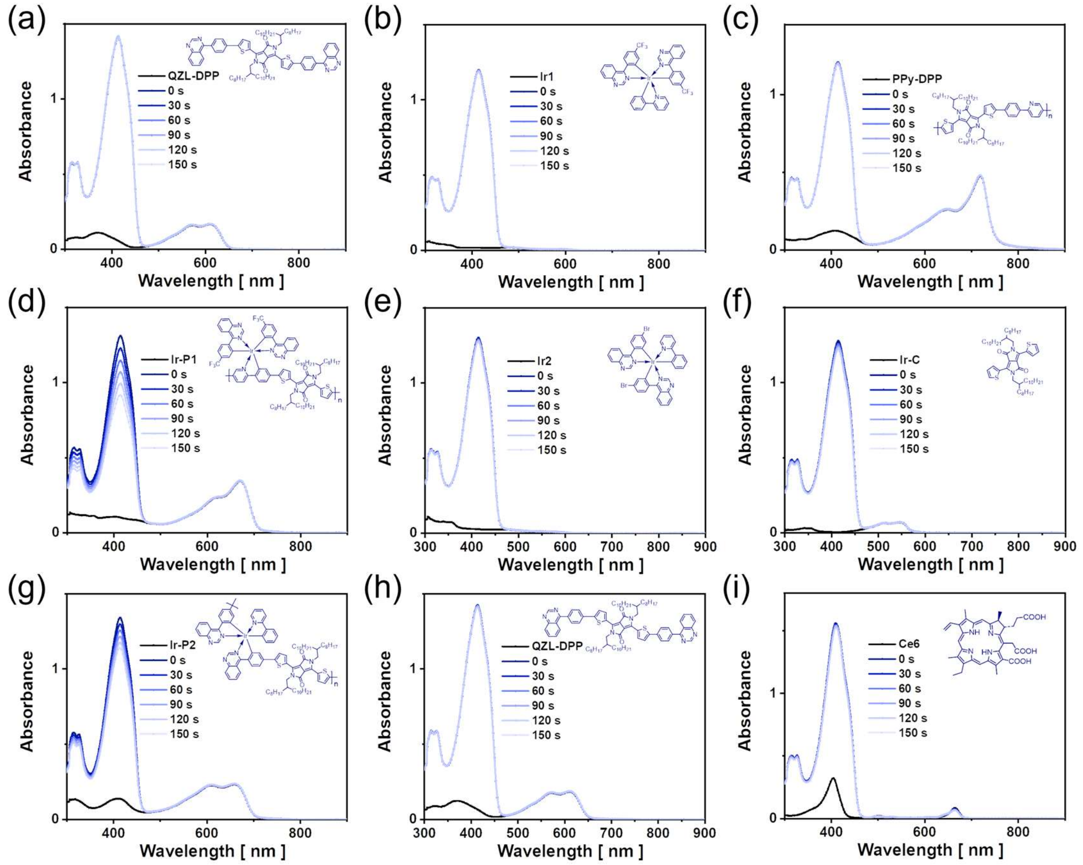


Supplementary Figure 8. The absorption spectra of DPBF in dichloromethane solutions. Experimental conditions: [DPBF] = 50 μM, [**QZL-DPP**] = [**Ir1**] = [**PPy-DPP**] = [**Ir-P1**] =[**Ir2**] = [**DPP**] = [**Ir-P2**] = [Ce6] = 2.8 μM with a 473 nm laser (5 mW·cm$^{-2}$) and recorded every 30 s. (a). **QZL-DPP** with a 680 nm laser (5 mW·cm$^{-2}$); (b). **Ir1**; (c). **PPy-DPP**; (d). **Ir-P1**; (e). **Ir2**; (f). **DPP**; (g). **Ir-P2**; (h). **QZL-DPP**; (i). Ce6.

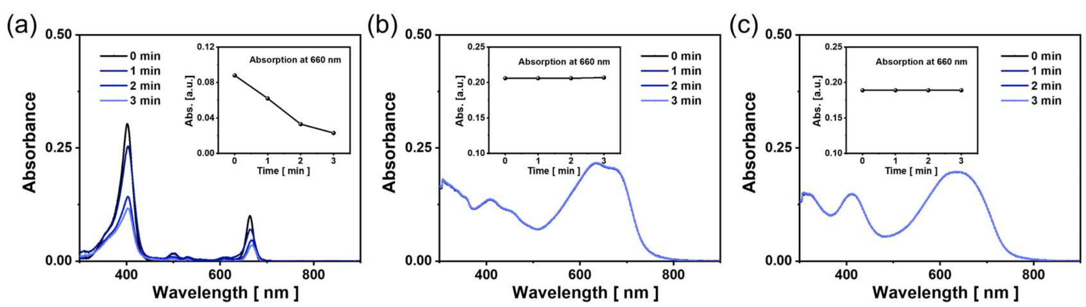


Supplementary Figure 9. Photostability of photosensitizers. (a). Ce6; (b). **MPdot1**; (c). **MPdot2**. Experimental condition: [Ce6] = [**MPdot1**] = [**MPdot2**] = 2.8 μM, irradiated with 680 nm laser (300 mW·cm$^{-2}$) and recorded every 1 min.

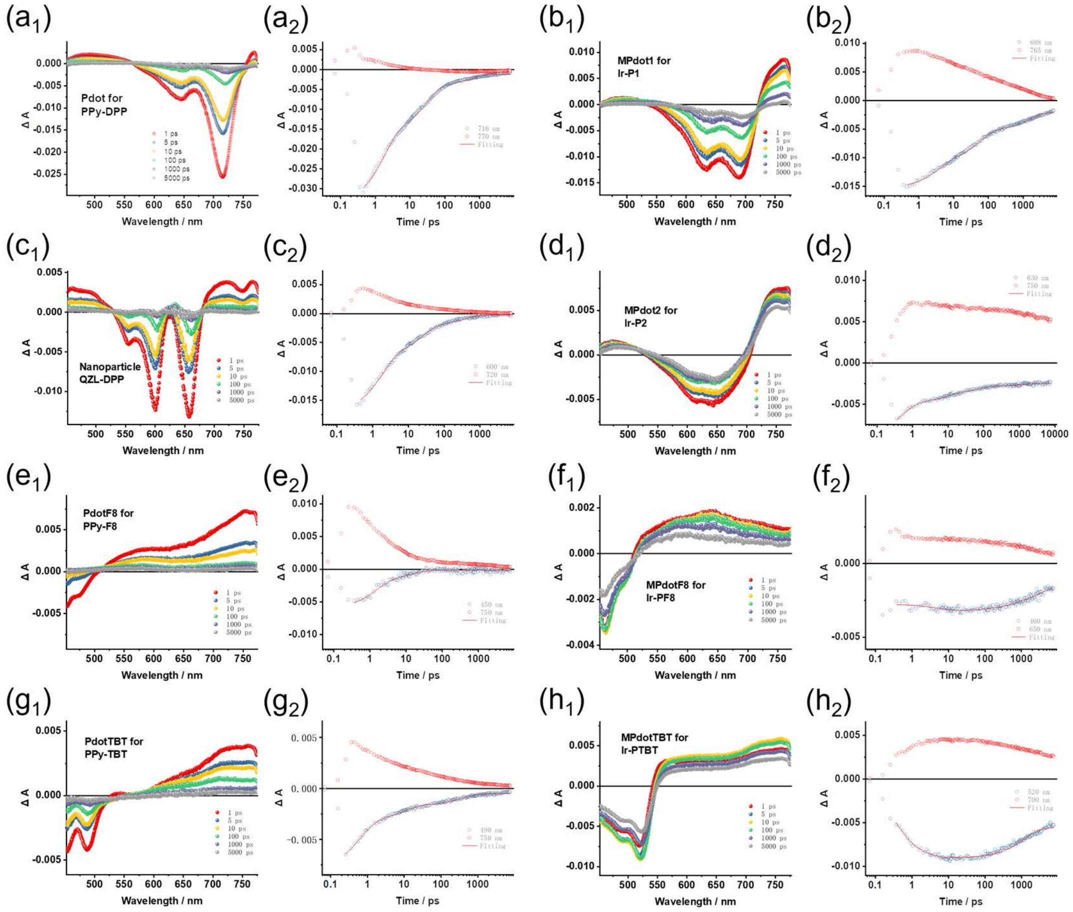


Supplementary Figure 10. Differential absorption spectra were obtained upon femtosecond pump probe experiments with time delays between 1 and 5000 ps. Time absorption profiles and corresponding fits at different wavelengths. ($a_1$, $a_2$). **PdotDPP** for **PPy-DPP**; ($b_1$, $b_2$). **MPdot1** for **Ir-P1**; ($c_1$, $c_2$). Nanoparticle for **QZL-DPP**; ($d_1$, $d_2$). **MPdot2** for **Ir-P2**; ($e_1$, $e_2$). **PdotF8** for **PPy-F8**; ($f_1$, $f_2$). **MPdotF8** for **Ir-PF8**; ($g_1$, $g_2$). **PdotTBT** for **PPy-TBT**; ($h_1$, $h_2$). **MPdotTBT** for **Ir-PTBT**.

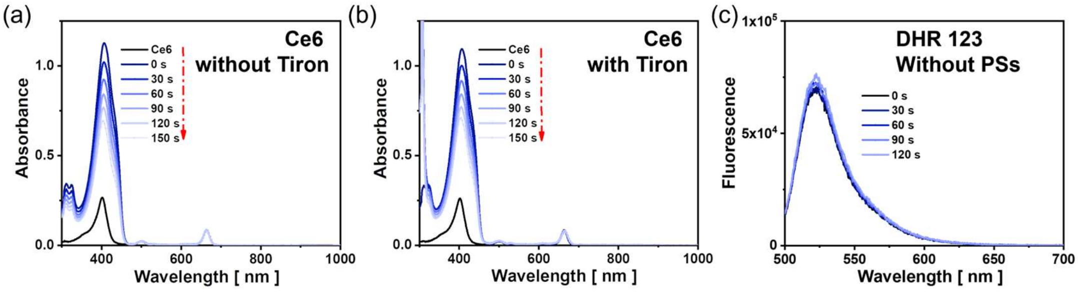


Supplementary Figure 11. Absorption spectra for DPBF in tetrahydrofuran/water (1:1, v/v) solution. Experimental conditions: [DPBF] = 50 μM, [Ce6] = 2.8 μM, [Tiron] with a 680 nm laser (20 mW·cm$^{-2}$)

and recorded every 30 s. (a). Ce6 and no Tiron; (b). Ce6 and Tiron. The fluorescence spectra for DHR 123 probes in water with a 680 nm laser (20 mW·cm$^{-2}$) and recorded every 30 s. Experimental conditions: [DHR 123] = 20 μM. (c). DHR 123 and no photosensitizer.

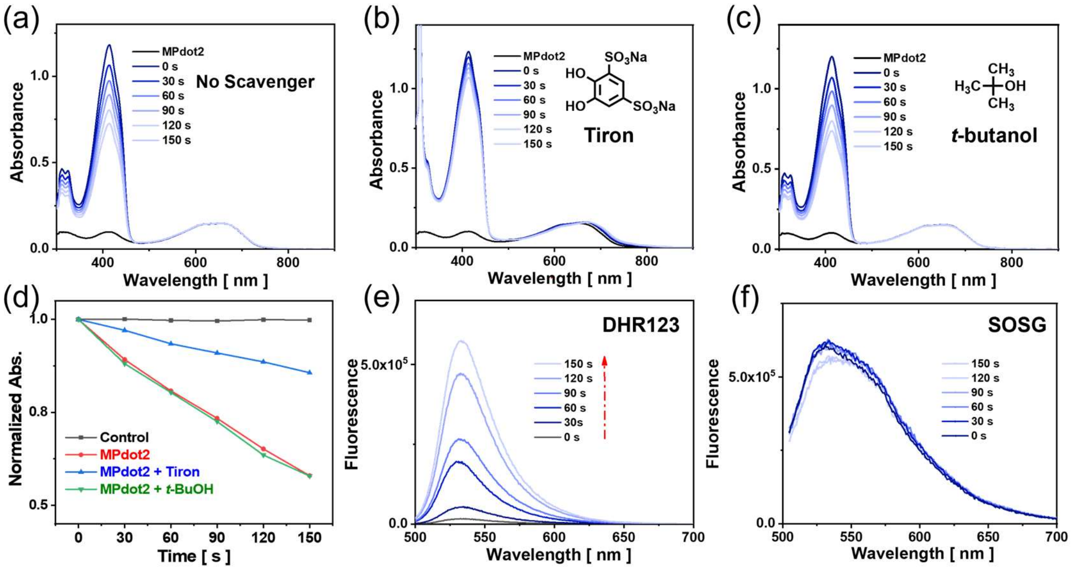


Supplementary Figure 12. Absorption spectra for DPBF in tetrahydrofuran/water (1:1, v/v) solution. Experimental conditions: [DPBF] = 50 μM, [**MPdot2**] = 2.8 μM, [Tiron] = [*t*-BuOH] = 50 mM with a 680 nm laser (20 mW·cm$^{-2}$) and recorded every 30 s. (a). no scavenger; (b). Tiron; (c). *t*-butanol, (d). The time-dependent normalized absorption was measured at 411 nm in the control group, MPdot group, and MPdot group with scavengers. The fluorescence spectra for ROS probes in water. Experimental conditions: [DHR 123] = [SOSG] = 20 μM, [**MPdot2**] = 2.8 μM with a 680 nm laser (20 mW·cm$^{-2}$). (e). DHR 123; (f). SOSG.

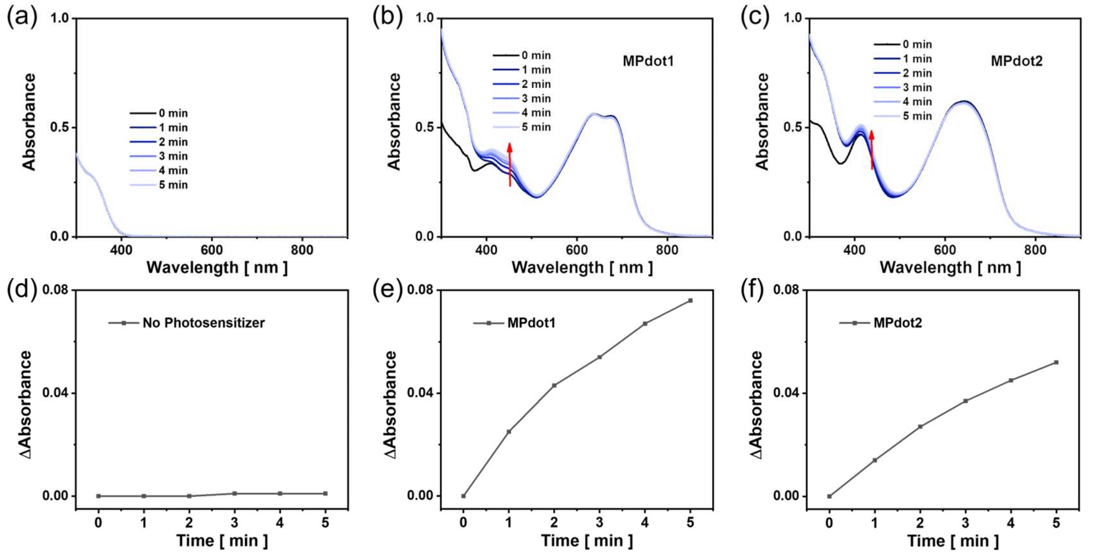


Supplementary Figure 13. The absorption spectra of WST-1 in aqueous solutions. Experimental conditions: [WST-1] = 50 μM, [**MPdot1**] = [**MPdot2**] = 14.0 μM with a 680 nm laser (20 mW·cm$^{-2}$) and recorded every 1 min. (a). no photosensitizer; (b). **MPdot1**; (c). **MPdot2**. And the incremental absorption at 435 nm: (d). no photosensitizer; (e). **MPdot1**; (f). **MPdot2**.

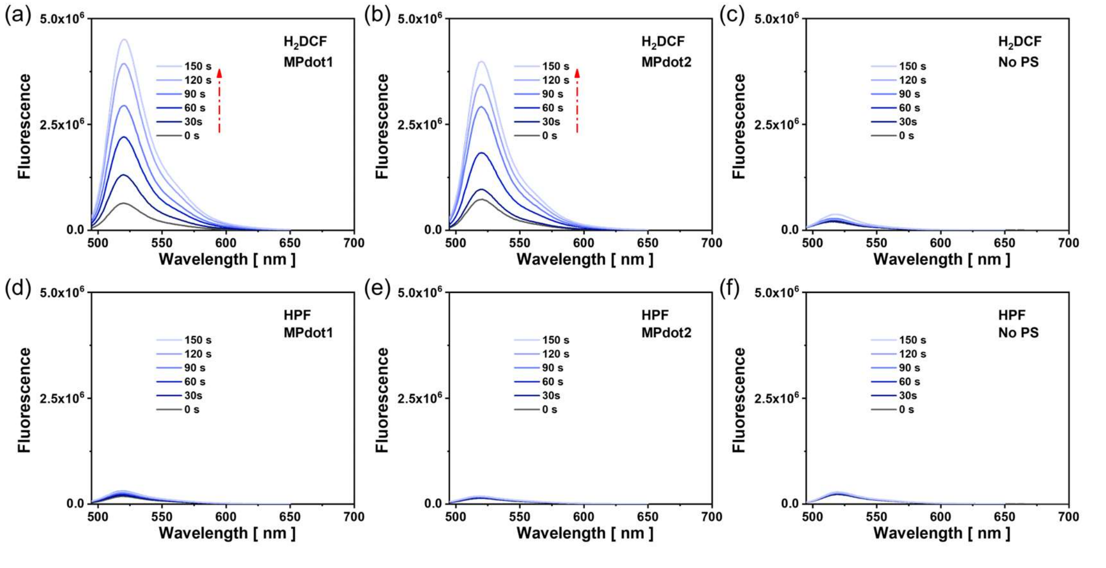


Supplementary Figure 14. The fluorescence spectra for ROS probes in water. $H_2DCF$ as probe, experimental conditions: [$H_2DCF$] = 20 μM, [**MPdot1**] = [**MPdot1**] = 2.8 μM with a 680 nm laser (20 mW·cm$^{-2}$). (a). **MPdot1**; (b). **MPdot2**; (c). No photosensitizer. HPF as probe, [HPF] = 20 μM. (d). **MPdot1**; (e). **MPdot2**; (f). No photosensitizer.

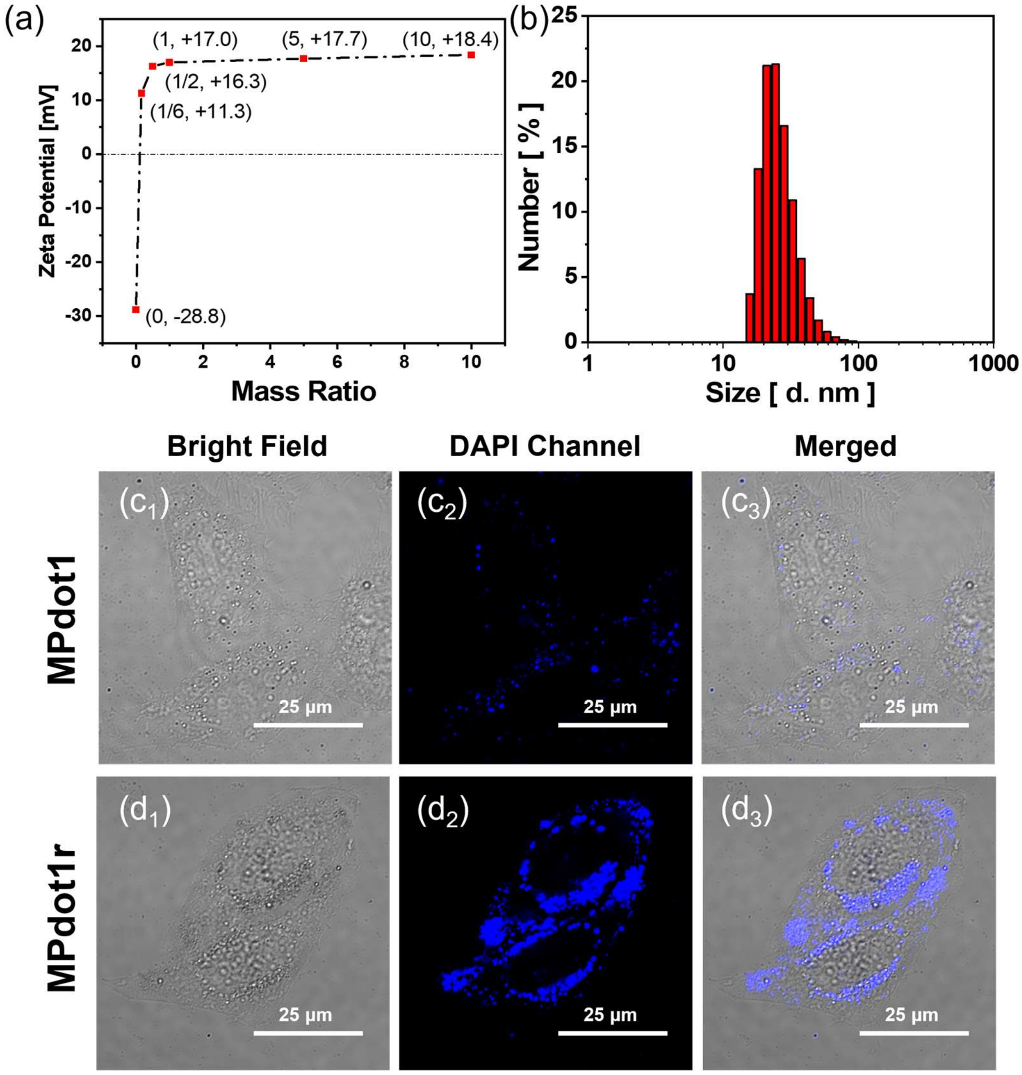


Supplementary Figure 15. Surface potential and size after post-decoration with R8 cell-penetrating peptide. (a). Mass ratio ($m_{\mathbf{R8}}$: $m_{\mathbf{MPdot1}}$) dependent surface potential of **MPdot1**. (b). Size of R8-decorated **MPdot1r** in water with a mass ratio of 1: 1. Cellular uptake assay of the R8 cell-penetrating peptide post-decorated MPdot. Experimental conditions: [**MPdot1r**] = 20 μg/mL incubated with cells for 20 hours ($c_1$, $c_2$, $c_3$). pristine **MPdot1** without R8 cell-penetrating peptide post-decoration; ($d_1$, $d_2$, $d_3$). **MPdot1r** with R8 cell-penetrating peptide post-decoration. All experiments were repeated three times independently with similar results.

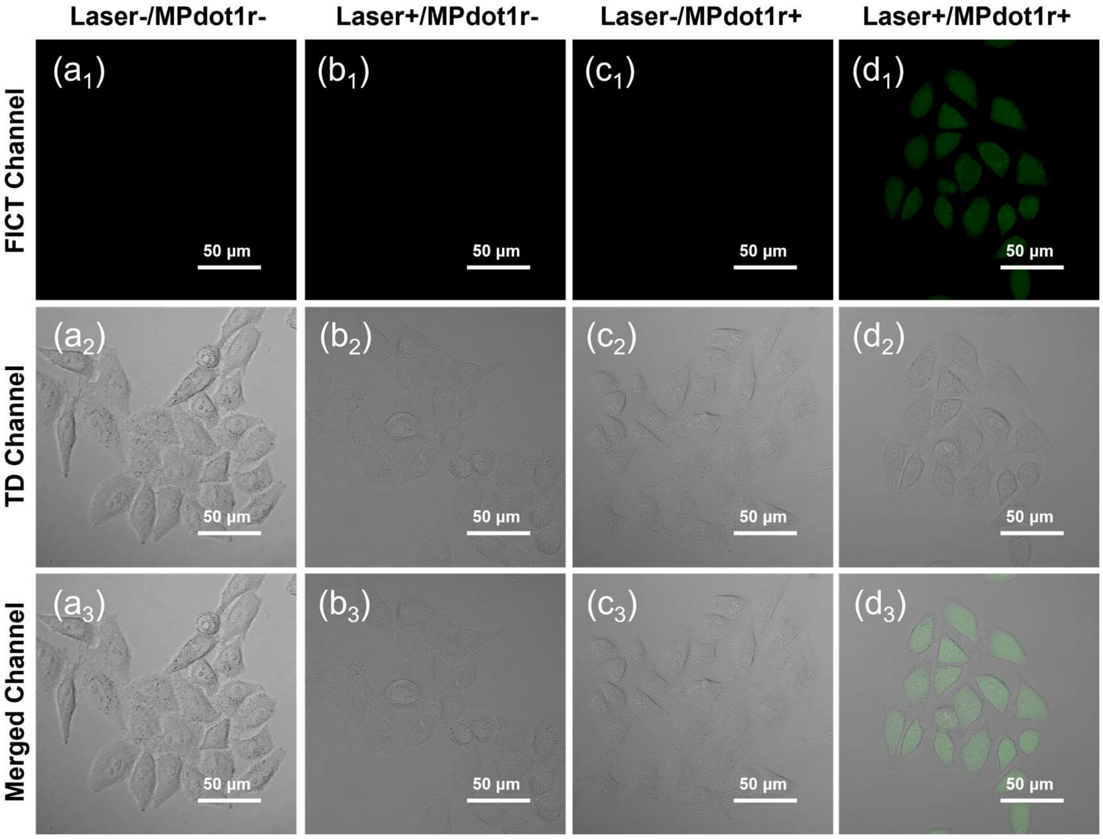


Supplementary Figure 16. Cellular ROS assay with $H_2DCFDA$. Experimental conditions: [**MPdot1r**] = 60 μg/mL incubated with cells for 20 hours, [$H_2DCFDA$] = 5 μM incubated with cells for 60 mins, 680 nm laser irradiation (40 mW·cm$^{-2}$) for 10 mins. ($a_1$, $a_2$, $a_3$). group without **MPdot1r** and without laser; ($b_1$, $b_2$, $b_3$). group without **MPdot1r** and with laser; ($c_1$, $c_2$, $c_3$). group with **MPdot1r** and without laser. ($d_1$, $d_2$, $d_3$). group **MPdot1r** and with laser. All experiments were repeated three times independently with similar results.

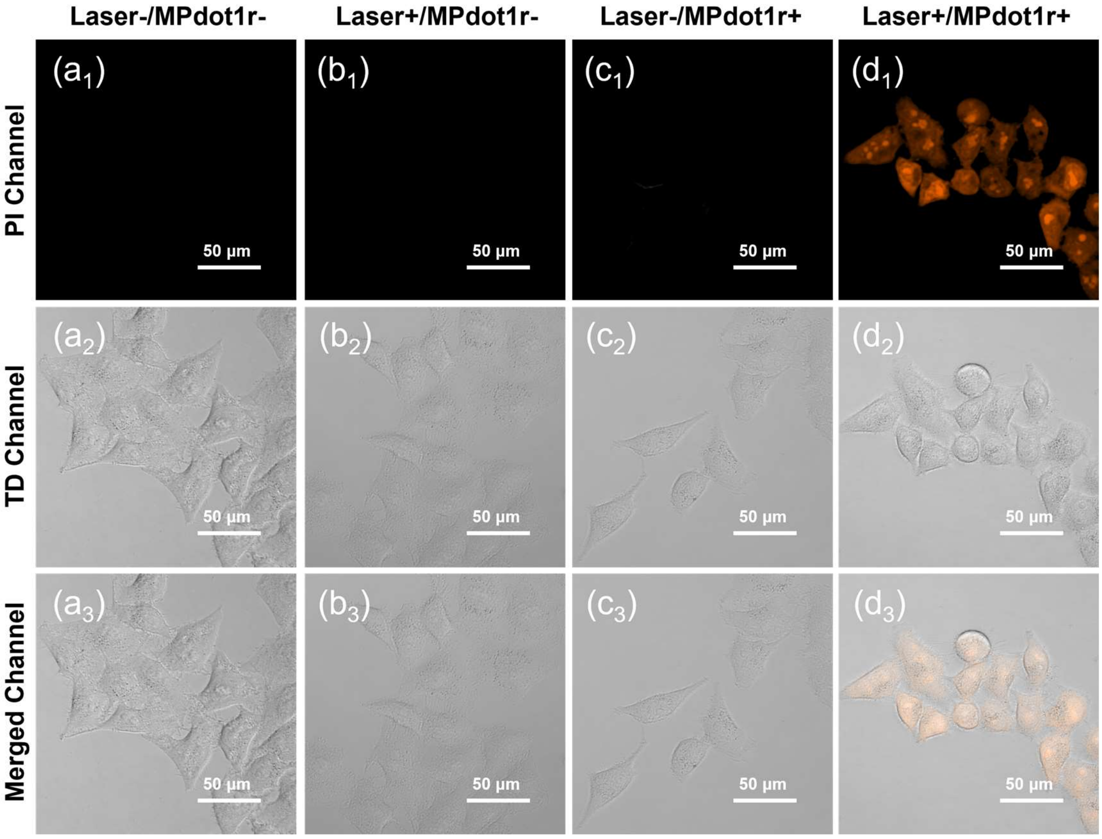


Supplementary Figure 17. $O_2^{\bullet-}$ assay with DHE. Experimental conditions: [**MPdot1r**] = 20 μg/mL incubated with cells for 20 hours, [DHE] = 5 μM incubated with cells for 30 mins, 680 nm laser irradiation (40 mW·cm$^{-2}$) for 5 mins. $O_2^{\bullet-}$ detection with DHE: ($a_1$, $a_2$, $a_3$). group without **MPdot1r** and without laser; ($b_1$, $b_2$, $b_3$). group without **MPdot1r** and with laser; ($c_1$, $c_2$, $c_3$). group with **MPdot1r** and without laser. ($d_1$, $d_2$, $d_3$). group **MPdot1r** and with laser. All experiments were repeated three times independently with similar results.

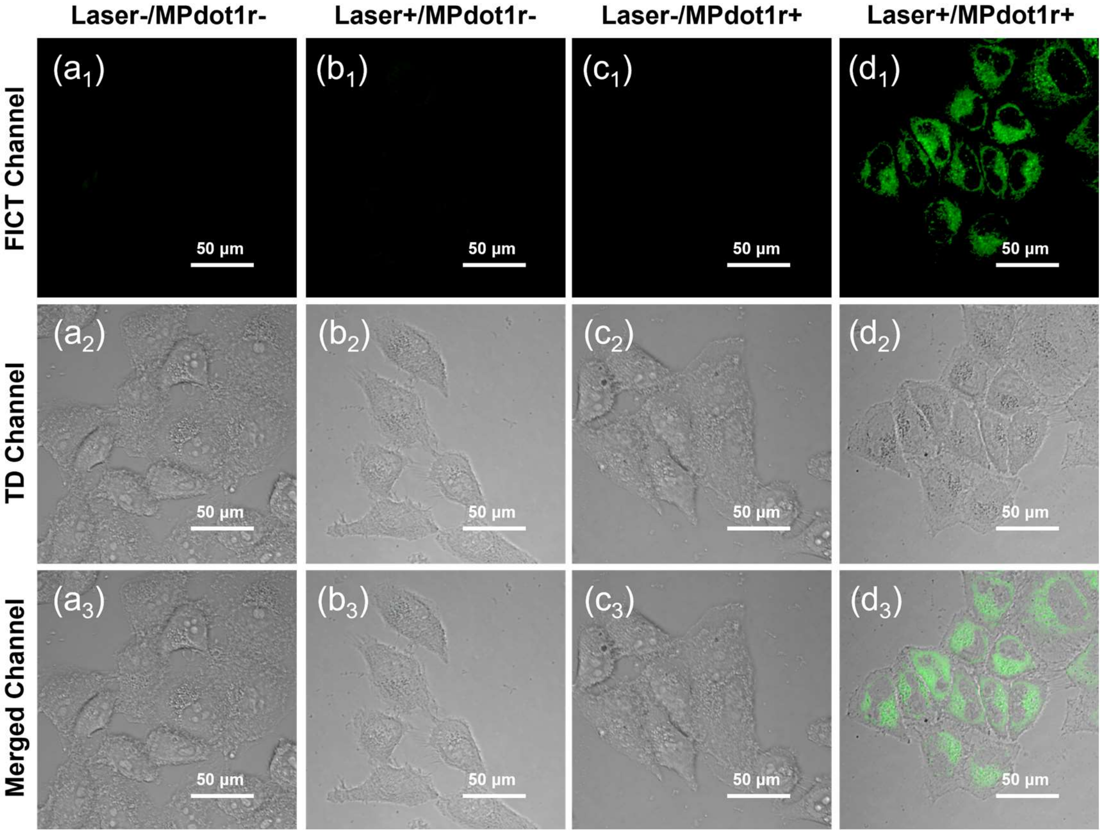


Supplementary Figure 18. $O_2^{\bullet-}$ assay with DHR 123. Experimental conditions: [**MPdot1r**] = 20 μg/mL incubated with cells for 20 hours, [DHR 123] = 5 μM incubated with cells for 30 mins, 680 nm laser irradiation (40 mW·cm$^{-2}$) for 5 mins. $O_2^{\bullet-}$ detection with DHR 123: ($a_1$, $a_2$, $a_3$). group without **MPdot1r** and without laser; ($b_1$, $b_2$, $b_3$). group without **MPdot1r** and with laser; ($c_1$, $c_2$, $c_3$). group with **MPdot1r** and without laser. ($d_1$, $d_2$, $d_3$). group **MPdot1r** and with laser. All experiments were repeated three times independently with similar results.

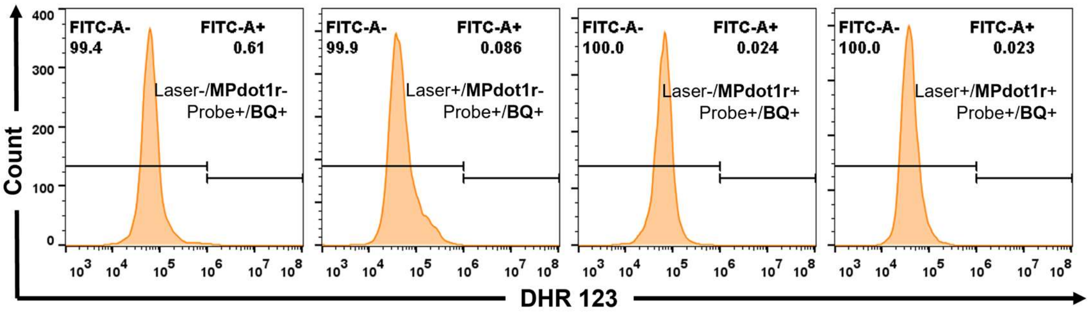

Supplementary Figure 19. Flow cytometry for $O_2^{\bullet-}$ detection with DHR 123. BQ: 1,4-benzoquinone (1 mM) used as an $O_2^{\bullet-}$ scavenger. All experiments were repeated three times independently with similar results.

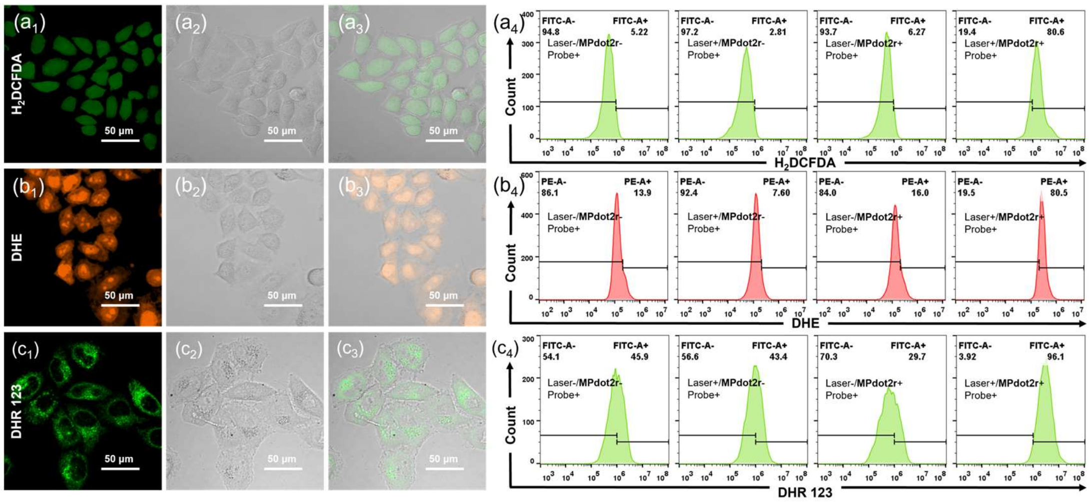


Supplementary Figure 20. ROS assay with $H_2DCFDA$, DHE, and DHR 123. Total ROS detection with $H_2DCFDA$ ($a_1$ - $a_4$). Experimental conditions: [**MPdot2r**] = 60 μg/mL with preincubation for 20 hours, [$H_2DCFDA$] = 5 μM with incubation for 60 mins, 680 nm laser irradiation (40 mW·cm$^{-2}$) for 10 mins. $O_2^{\bullet-}$ detection with DHE ($b_1$ - $b_4$). Experimental conditions: [**MPdot2r**] = 20 μg/mL with preincubation for 20 hours, [DHE] = 5 μM with incubation for 30 mins, 680 nm laser irradiation (40 mW·cm$^{-2}$) for 5 mins. $O_2^{\bullet-}$ detection with DHR 123 ($c_1$ - $c_4$). Experimental conditions: [**MPdot2r**] = 20 μg/mL with preincubation for 20 hours, [DHR 123] = 5 μM with incubation for 30 mins, 680 nm laser irradiation (40 mW·cm$^{-2}$) for 5 mins.($a_1$, $b_1$, and $c_1$). The FICT channel; ($a_2$, $b_2$, and $c_2$). The transmitted light differential interference (TD) channel; ($a_3$, $b_3$, and $c_3$). The merged channel. ($a_4$, $b_4$, and $c_4$). Flow cytometry for ROS generation. All experiments were repeated three times independently with similar results.

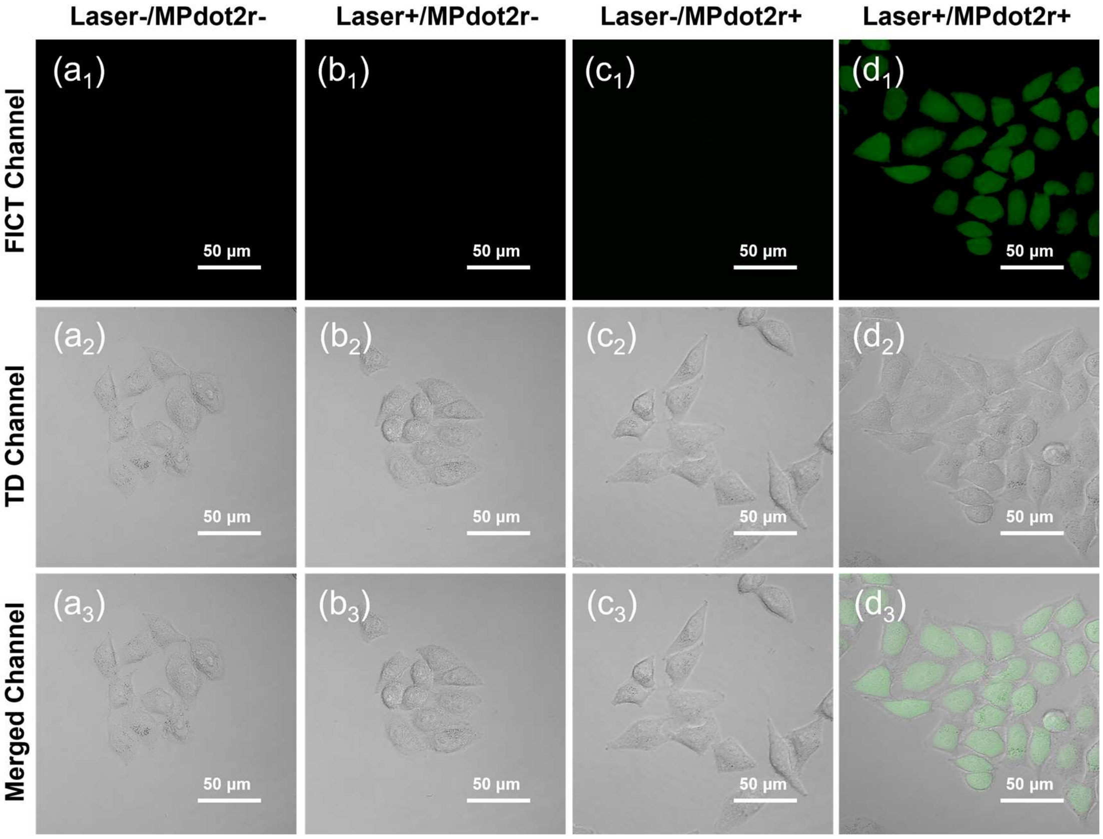


Supplementary Figure 21. Cellular ROS assay with $H_2DCFDA$. Experimental conditions: [**MPdot2r**] = 60 μg/mL incubated with cells for 20 hours, [$H_2DCFDA$] = 5 μM incubated with cells for 60 mins, 680 nm laser irradiation (40 mW·cm$^{-2}$) for 10 mins. ($a_1$, $a_2$, $a_3$). group without **MPdot2r** and without laser; ($b_1$, $b_2$, $b_3$). group without **MPdot2r** and with laser; ($c_1$, $c_2$, $c_3$). group with **MPdot2r** and without laser. ($d_1$, $d_2$, $d_3$). group **MPdot2r** and with laser. All experiments were repeated three times independently with similar results.

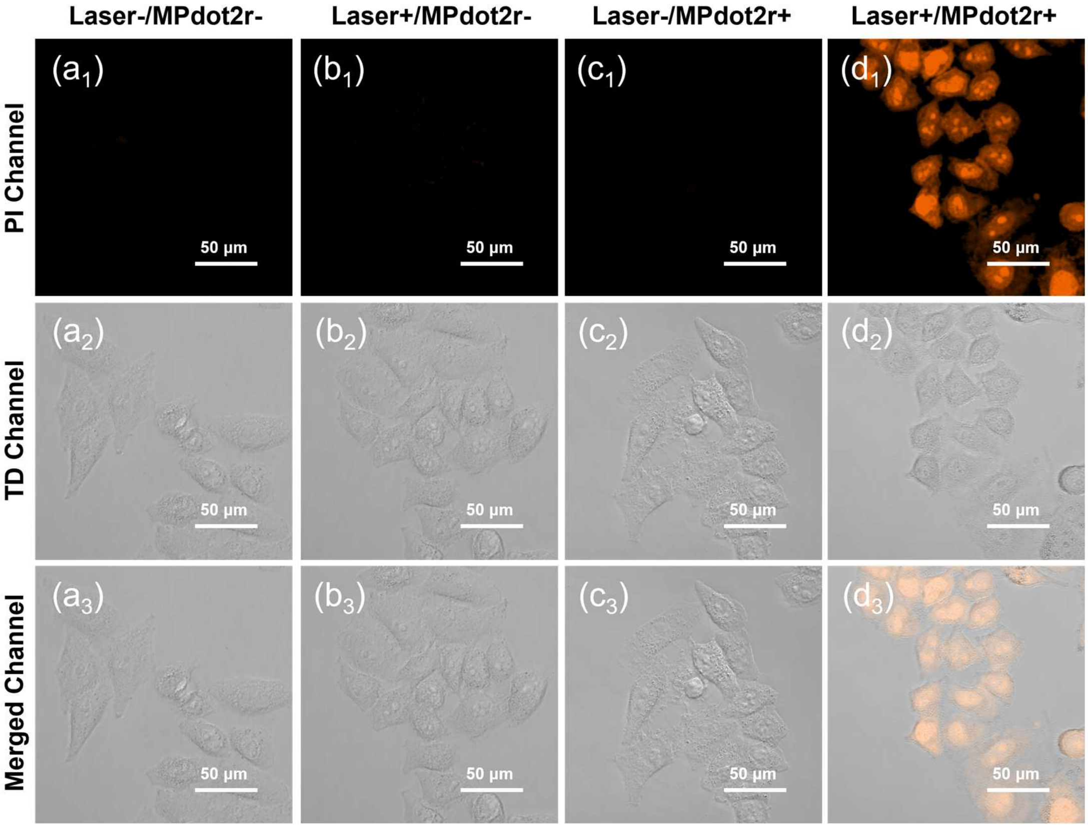


Supplementary Figure 22. $O_2^{\bullet-}$ assay with DHE. Experimental conditions: [**MPdot2r**] = 20 μg/mL incubated with cells for 20 hours, [DHE] = 5 μM incubated with cells for 30 mins, 680 nm laser irradiation (40 mW·cm$^{-2}$) for 5 mins. $O_2^{\bullet-}$ detection with DHE: ($a_1$, $a_2$, $a_3$). group without **MPdot2r** and without laser; ($b_1$, $b_2$, $b_3$). group without **MPdot2r** and with laser; ($c_1$, $c_2$, $c_3$). group with **MPdot2r** and without laser. ($d_1$, $d_2$, $d_3$). group **MPdot2r** and with laser. All experiments were repeated three times independently with similar results.

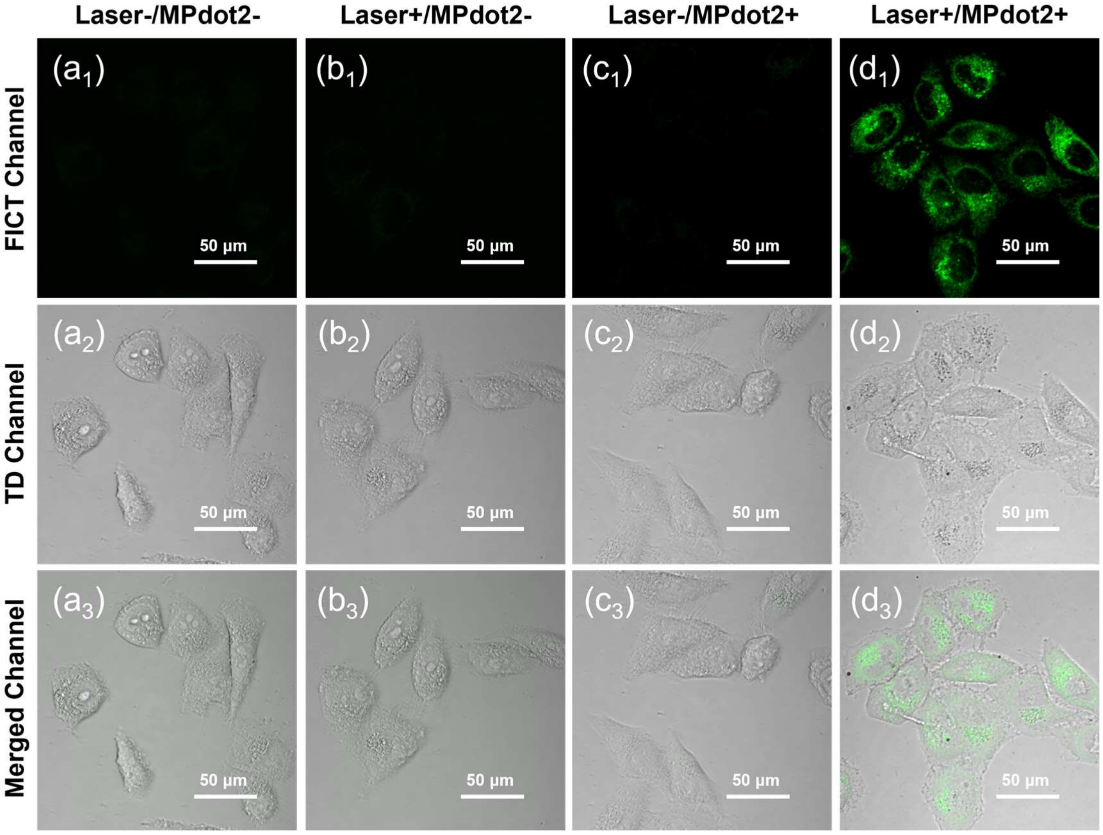


Supplementary Figure 23. $O_2^{\bullet-}$ assay with DHR 123. Experimental conditions: [**MPdot2r**] = 20 μg/mL incubated with cells for 20 hours, [DHR 123] = 5 μM incubated with cells for 30 mins, 680 nm laser irradiation (40 mW·cm$^{-2}$) for 5 mins. $O_2^{\bullet-}$ detection with DHR 123: ($a_1$, $a_2$, $a_3$). group without **MPdot2r** and without laser; ($b_1$, $b_2$, $b_3$). group without **MPdot2r** and with laser; ($c_1$, $c_2$, $c_3$). group with **MPdot2r** and without laser. ($d_1$, $d_2$, $d_3$). group **MPdot2r** and with laser. All experiments were repeated three times independently with similar results.

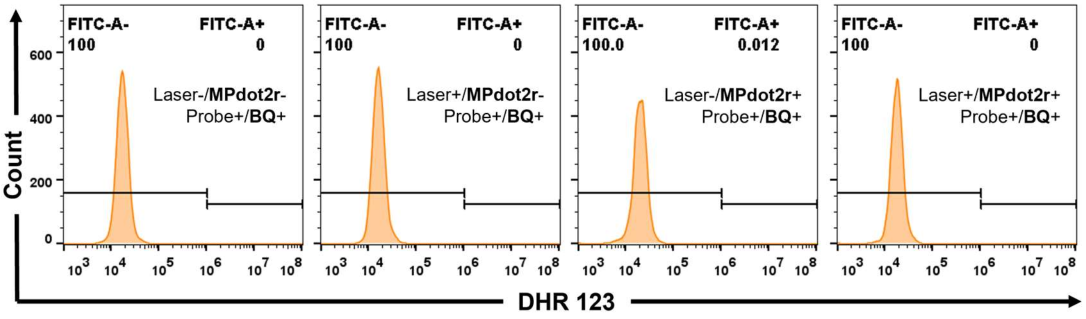

Supplementary Figure 24. Flow cytometry for $O_2^{\bullet-}$ detection with DHR 123. BQ: 1,4-benzoquinone (1 mM) used as an $O_2^{\bullet-}$ scavenger. All experiments were repeated three times independently with similar results.

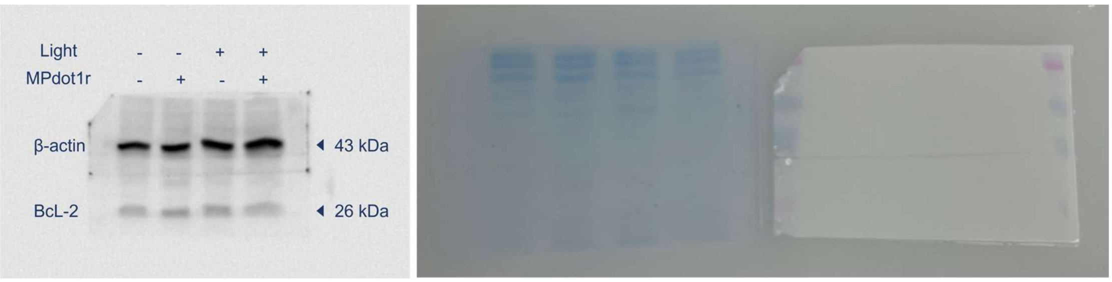


Supplementary Figure 25. Unprocessed and full scan of gel and membrane for Western Blot. All experiments were repeated three times independently with similar results.

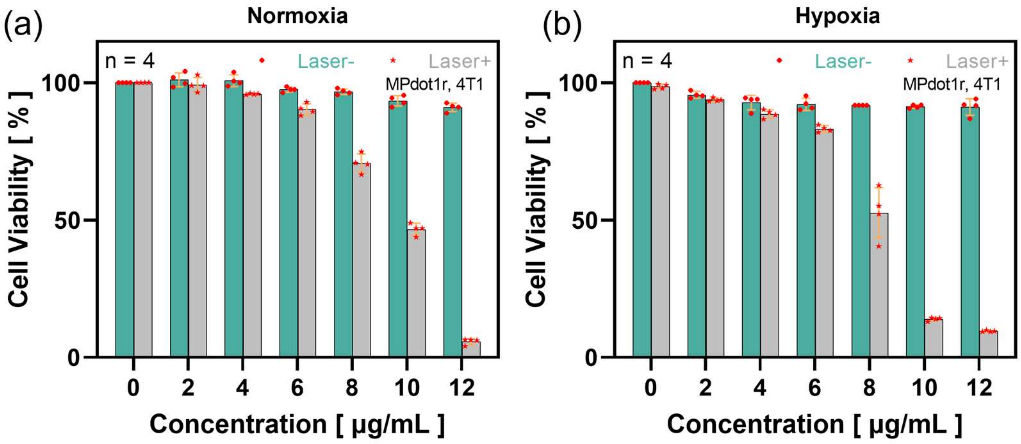


Supplementary Figure 26. Cell viability was assayed with Cell Counting Kit-8. Experimental conditions: 4T1 cell was preincubated with **MPdot1r** for 20 hours before 680 nm laser irradiation (100 mW·cm$^{-2}$) for 10 mins. (a). normoxia ($V\%_{O2}$ = 21%) and (b). hypoxia ($V\%_{O2}$ = 1%), respectively.

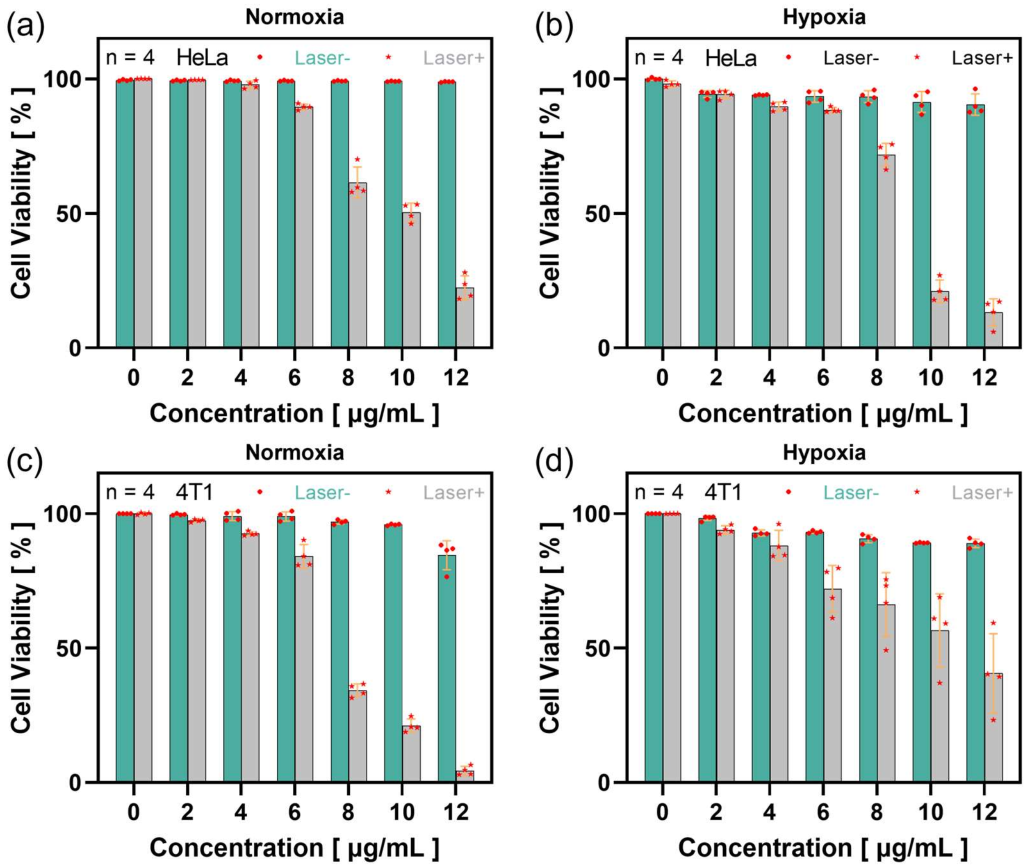


Supplementary Figure 27. Cell viability was assayed with Cell Counting Kit-8. Experimental conditions: HeLa and 4T1 cell was preincubated with **MPdot2r** for 20 hours before 680 nm laser irradiation (100 mW·cm$^{-2}$) for 10 mins. (a). HeLa, normoxia ($V\%_{O2}$ = 21%), and (b). HeLa, hypoxia ($V\%_{O2}$ = 1%); (c). 4T1, normoxia ($V\%_{O2}$ = 21%), and (d). 4T1, hypoxia ($V\%_{O2}$ = 1%), respectively.

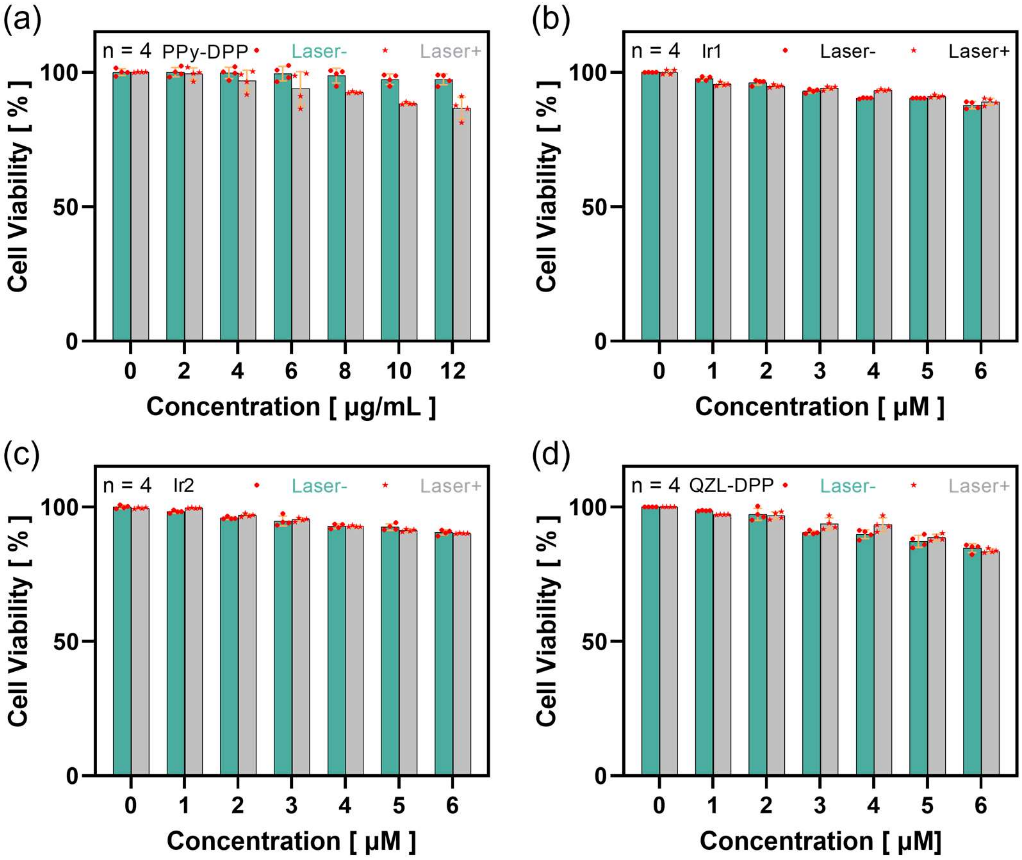


Supplementary Figure 28. Cell viability was assayed with Cell Counting Kit-8. Experimental conditions: HeLa photosensitizers for 20 hours before 680 nm laser irradiation (100 mW·cm$^{-2}$) for 10 mins. (a). **Pdotr** of **PPy-DPP**; (b). **Ir1**; (c). **Ir2**; (d). **QZL-DPP**, respectively.

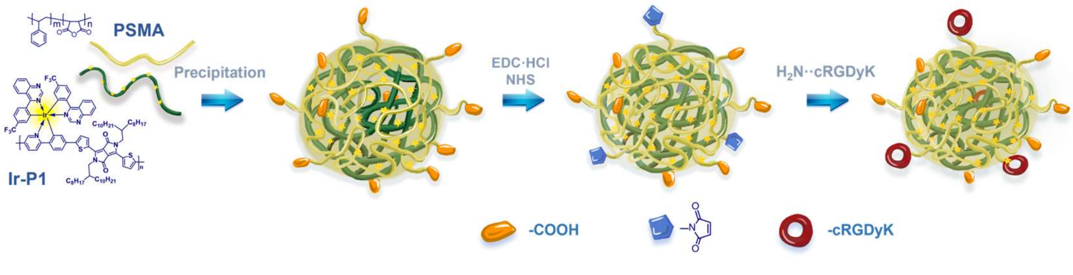


Supplementary Figure 29. Illustration of fabrication and post-bioconjugation of **MPdot1c**.

**Supplementary Table 3. Photoacoustic Characterization Parameters**

| Vevo LAZR system | Test | Model | Acquisition | | | | | | | | | | |
|---|---|---|---|---|---|---|---|---|---|---|---|---|---|
| | | | Wavelength Range | Frame Rate | PA Gain | 2D Gain | Depth | Width | Sensitivity | Resp Gate | Persistence | PA Acquisition | Wavelength [nm] | Correct Energy |
| | Materials in Solution | Single | 680-970 | 5 | 40.0 | 29.0 | 10.0 | 12.08 | High | Off | Low | Single | 680 | On |
| | Accumulation in vivo | Single | 680-970 | 5 | 41.0 | 29.0 | 10.0 | 12.08 | High | Off | Low | Single | 680 | On |
| | Oxygenation | Oxy-Hemo | 680-970 | - | 40.0 | 29.0 | 10.0 | 12.08 | High | Off | Low | $sO_2$/HbT | 750/850 | On |

| Vevo LAZR system | Test | Model | Transmit | | | Display | | | | | | | |
|---|---|---|---|---|---|---|---|---|---|---|---|---|---|
| | | | Frequency [MHz] | 2D Power [%] | PA Power [%] | Display Type | Display Map | Priority [%] | Threshold HbT | Brightness | Contrast | PA Brightness | PA contrast |
| | Materials in Solution | Single | 40 | 100 | 100 | - | PA1 | 99 | - | 50 | 50 | 55 | 80 |
| | Accumulation in vivo | Single | 40 | 100 | 100 | - | PA1 | 99 | - | 50 | 50 | 59 | 80 |
| | Oxygenation | Oxy-Hemo | 40 | 100 | 100 | OxyZated | PA2 | 99 | 13 | 44 | 41 | - | - |

Supplementary Table 3. Photoacoustic characterization parameters.

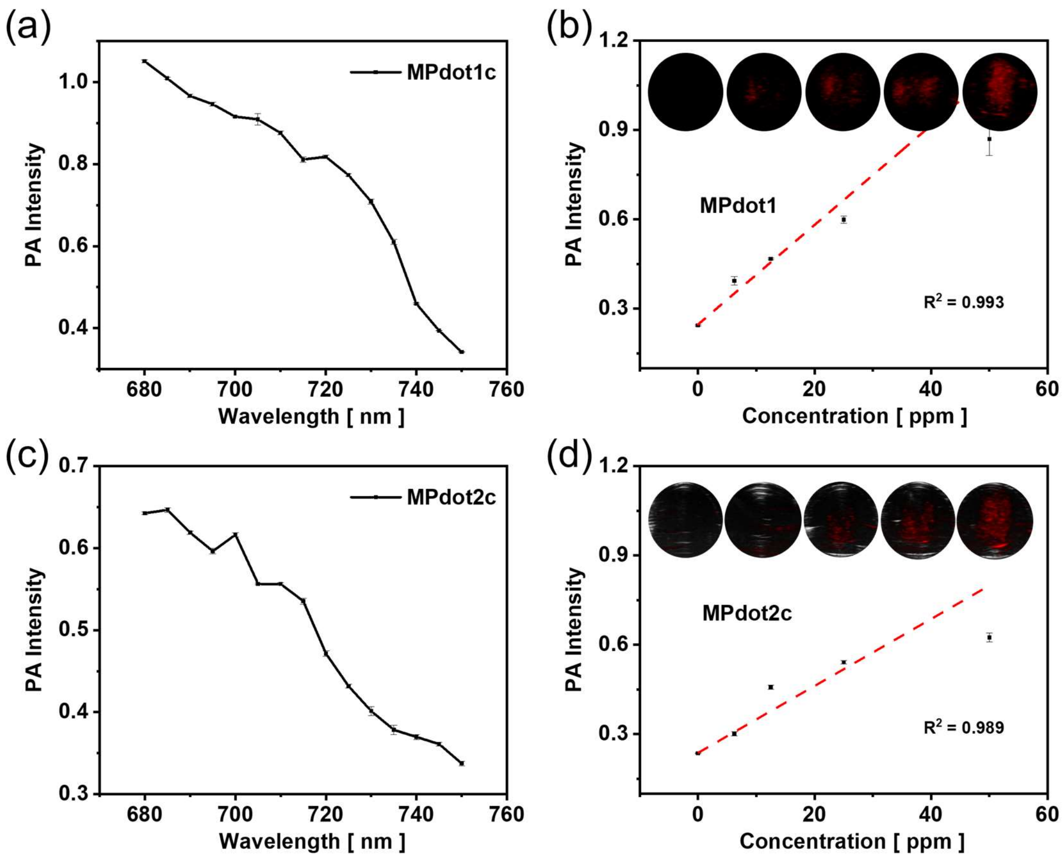


Supplementary Figure 30. Photoacoustic spectra for MPdots. (a, b) **MPdot1c**; (c,d) **MPdot2c**. Experimental condition: for photoacoustic spectra, the photoacoustic signal was acquired at 680, 685, 690, …, 745, 750 nm (100 ppm **MPdot1c** or 50ppm **MPdot2c** in PBS, PA-Model(single), 100% power, 40-dB gain, 40-MHz frequency). For concentration-dependent photoacoustic spectra, the photoacoustic

signal was acquired at 0, 6.25, 12.5, 25, and 50 ppm of **MPdot1c** or **MPdot2c** in PBS (680 nm, PA-Model(single), 100% power, 40-dB gain, 40-MHz frequency).

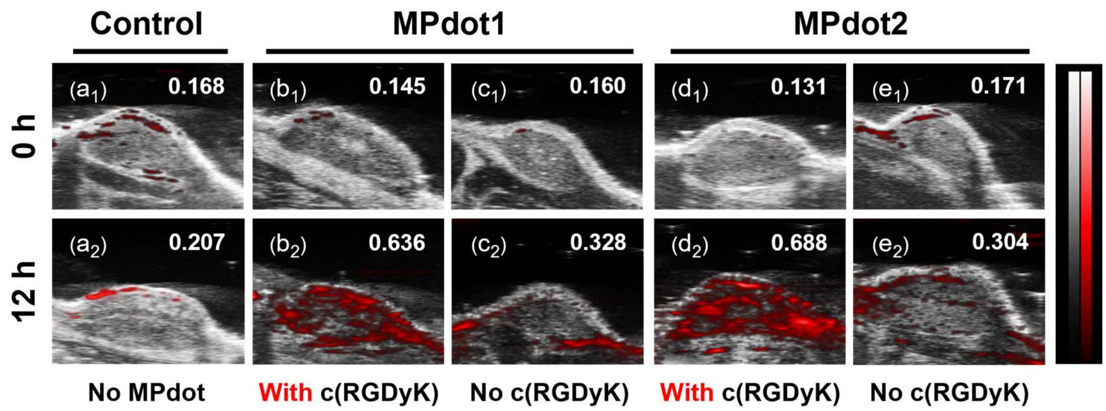


Supplementary Figure 31. PA images of mice with different metallopolymeric nanoparticles. The pictures were taken at 0 and 12 hours after MPdot administration (400 pp, 100 μL). ($a_1$, $a_2$). Control without any MPdot; ($b_1$, $b_2$). **MPdot1** with c(RGDyK) modification; ($c_1$, $c_2$). **MPdot1** with no modification; ($d_1$, $d_2$). **MPdot2** with c(RGDyK) modification; ($e_1$, $e_2$). **MPdot2** with no modification. Experimental condition: 680 nm, PA-Model(single), 100% power, 40-dB gain, 40-MHz frequency. The intensity of the photoacoustic signal in the tumor region was determined by delineating the region of interest by the imaging system.

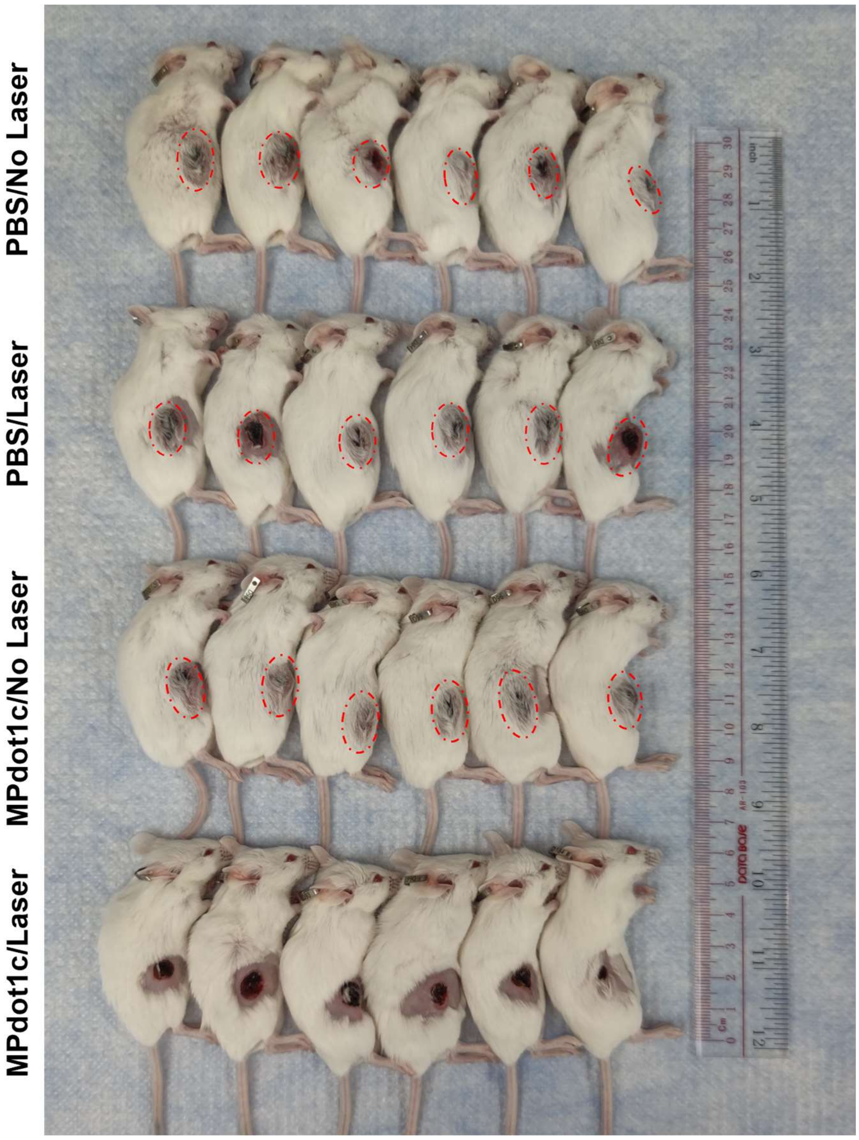


Supplementary Figure 32. The mice after the treatment (on the 17$^{th}$ day).

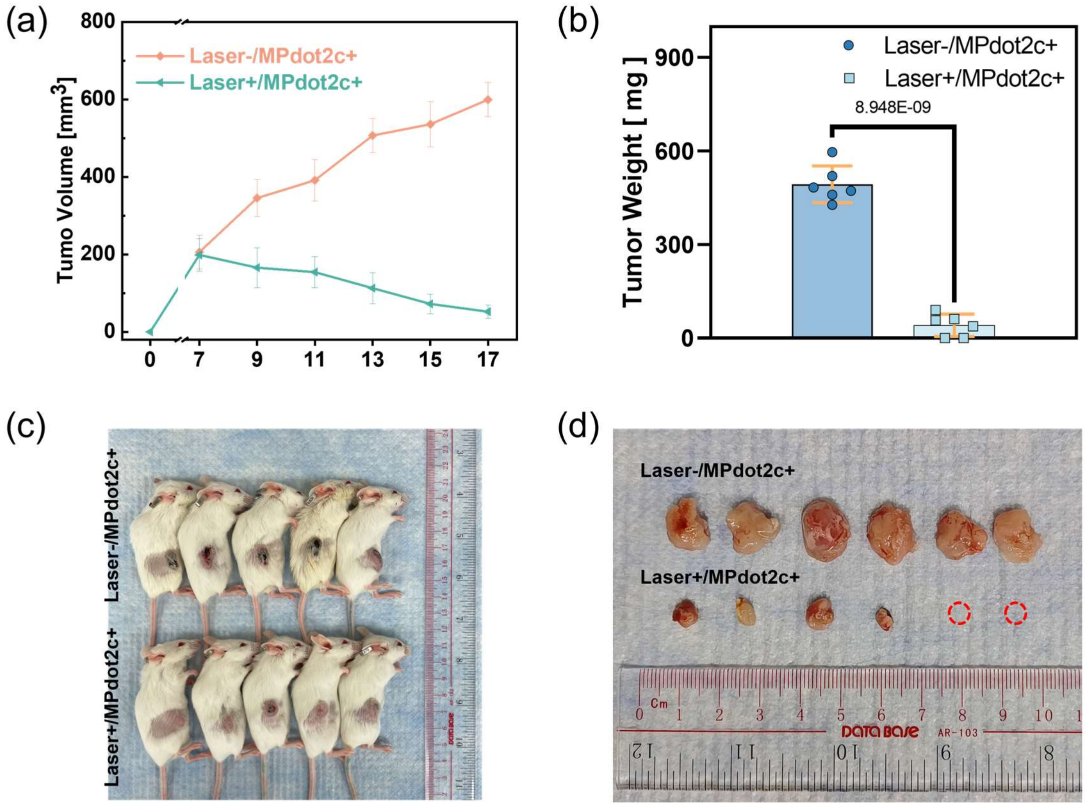


Supplementary Figure 33. (a). Tumor volume was measured at the indicated day with a caliper and calculated using the following equation: $V = \frac{\pi}{6} \times A \times B^2$, $A$ is long diameter and $B$ is short diameters of the tumor. (b). Tumor weight measured after dissection. All tumor weights were acquired from individual mice in each group. (c). Mice photo and (d). Tumor photo for mice in each group.

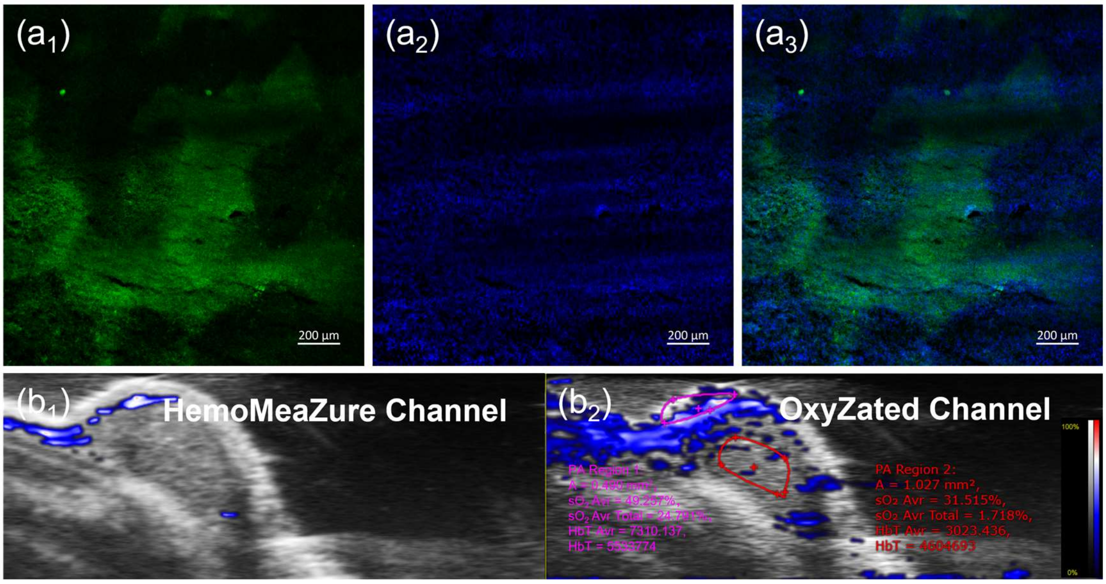

Supplementary Figure 34. Hypoxia detection in tumor tissue. Immunofluorescence staining with Hpoxyprobe$^{TM}$-1 Green Kit. ($a_1$). FITC channel; ($a_2$). DAPI; ($a_3$). Merged channel. Photoacoustic imaging on Vevo LAZR-X. ($b_1$). HemoMeaZure Channel; ($b_2$). OxyZated Channel. sO2 and HbT in the epidermal area (Region1) and tumor area (Region 2) were measured using the Vevo LAZR imaging system to delineate the area of interest (40-dB gain, 10.00 mm depth, 12.08 mm width).

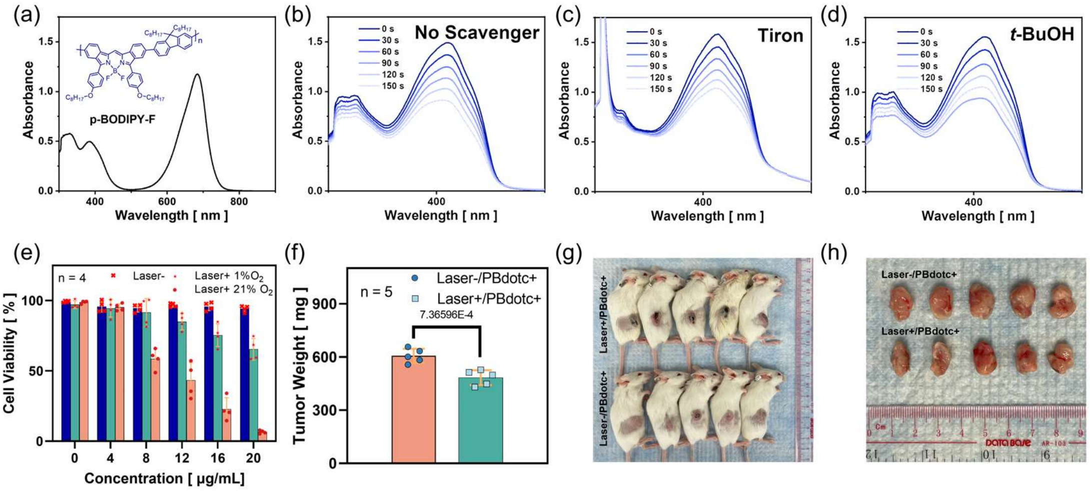


Supplementary Figure 35. **p-BODIPY-F**'s photochemical properties study and therapeutic application *in vitro* and *in vivo*. (a). Chemical structure of **p-BODIPY-F** and absorption spectra of **PBdot** (10 ppm, 9.18 μM). ROS generation without or in the presence of scavengers. (b). No scavenger; (c). Tiron as $O_2^{\bullet -}$ scavenger; (d). *t*-BuOH as $OH^{\bullet}$ scavenger. Experimental conditions: [DPBF] = 50 μM, [**PBdot**] = 10 ppm = 9.18 μM, [Tiron] = [*t*-BuOH] = 50 mM with a 680 nm laser (100 $mW \cdot cm^{-2}$) and recorded every 30 s. (e). HeLa cell viability assay with varying concentrations of Pdotr: blue column, cells without laser treatment; green column, cells with laser treatment in hypoxia ($V\%O_2$ = 1%) and orange column, cells with laser treatment in normoxia ($V\%O_2$ = 21%), respectively. Experimental condition: **PBdotr** incubation duration, 20 hours; laser treatment, a 680 nm laser at a power of 200 $mW \cdot cm^{-2}$ for 20 mins. (f). Tumor weight measured after dissection. Tumor weights were acquired from individual mice in groups. (g). Mice photo and (h). Tumor photo for mice in each group. Experimental condition: Pdot administration, 100 μL of c(RGDyK)-modified **PBdotc** at a concentration of 400 μg/mL; laser treatment, 680 nm laser with a power of 400 $mW \cdot cm^{-2}$ for 15 mins.

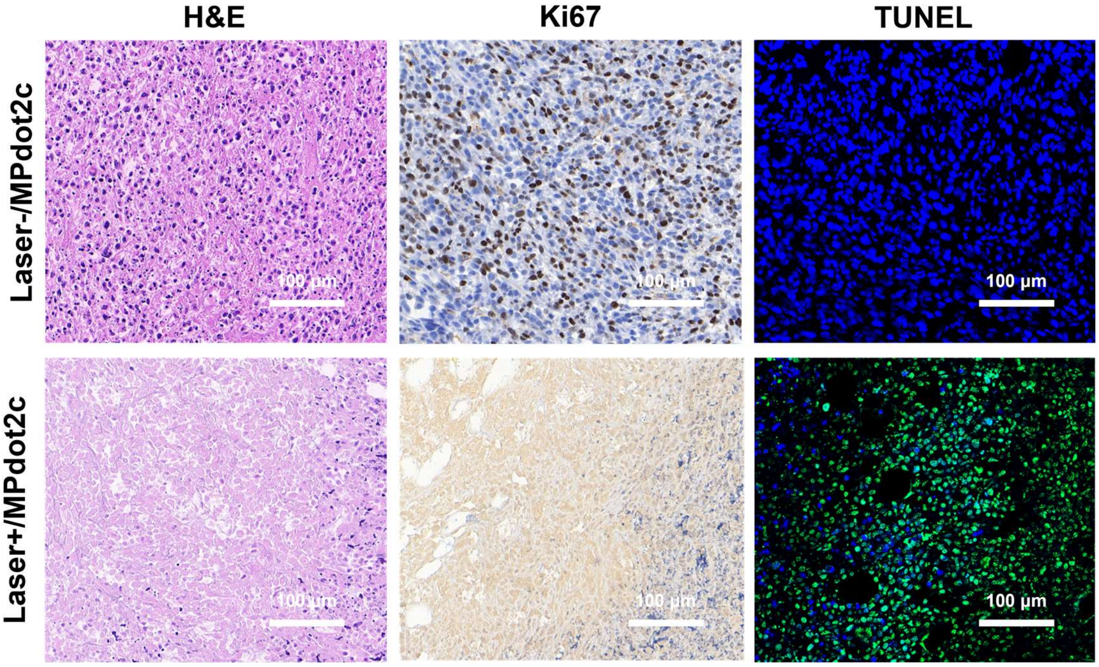


Supplementary Figure 36. H&E staining, Ki67 immunohistochemistry, and TUNEL staining of tumor sections for Laser+/**MPdot2c** and Laser+/**MPdot2c** groups. N = 3 tissue slices were prepared independently and observed with similar results.

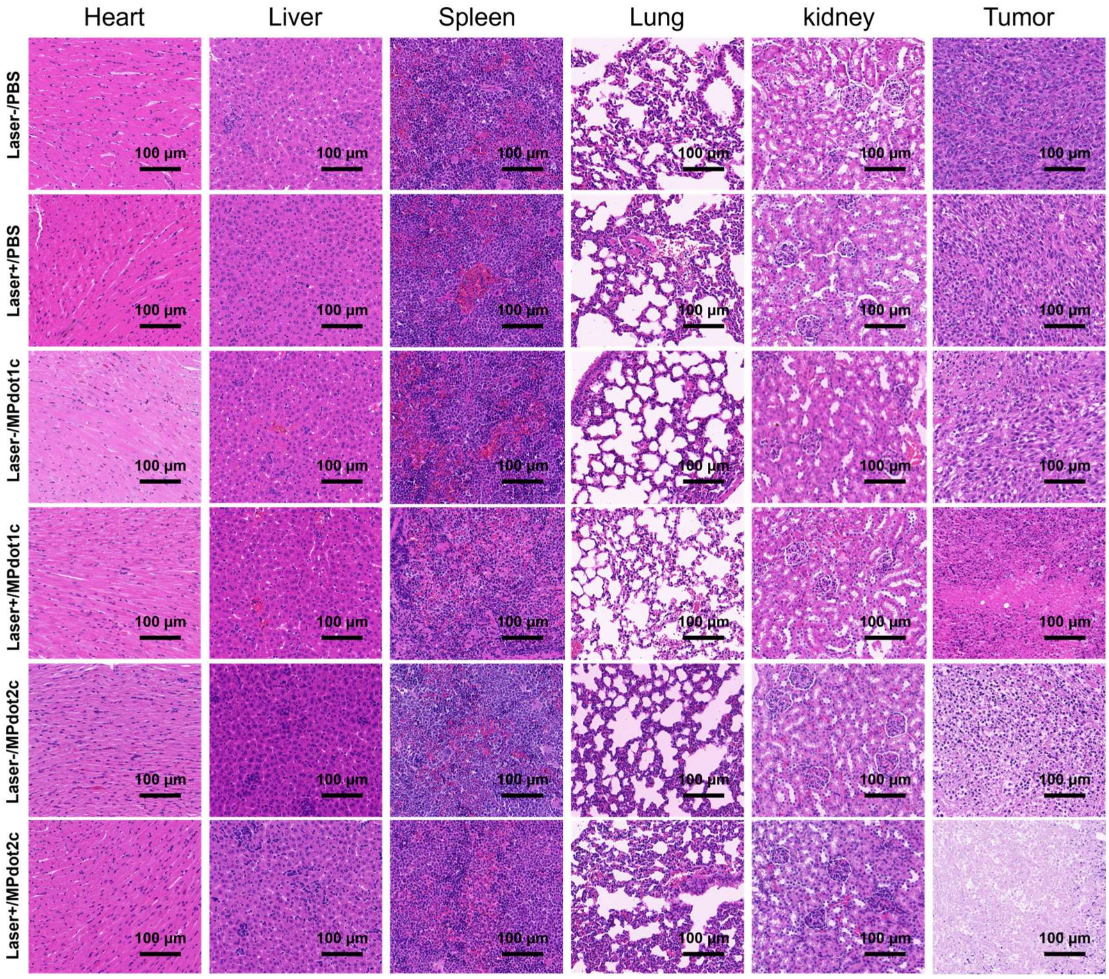


Supplementary Figure 37. Hematoxylin and eosin (H&E) staining for tumor/heart/liver /spleen/lung/kidney inLaser-/PBS, Laser+/PBS, Laser-/**MPdot1c**, Laser+/**MPdot1c**, Laser-/**MPdot2c**, and Laser+/**MPdot2c** group. N = 3 tissue slices were prepared independently and observed with similar results.

## $^{1}$HNMR, $^{13}$CNMR, and Mass Spectra

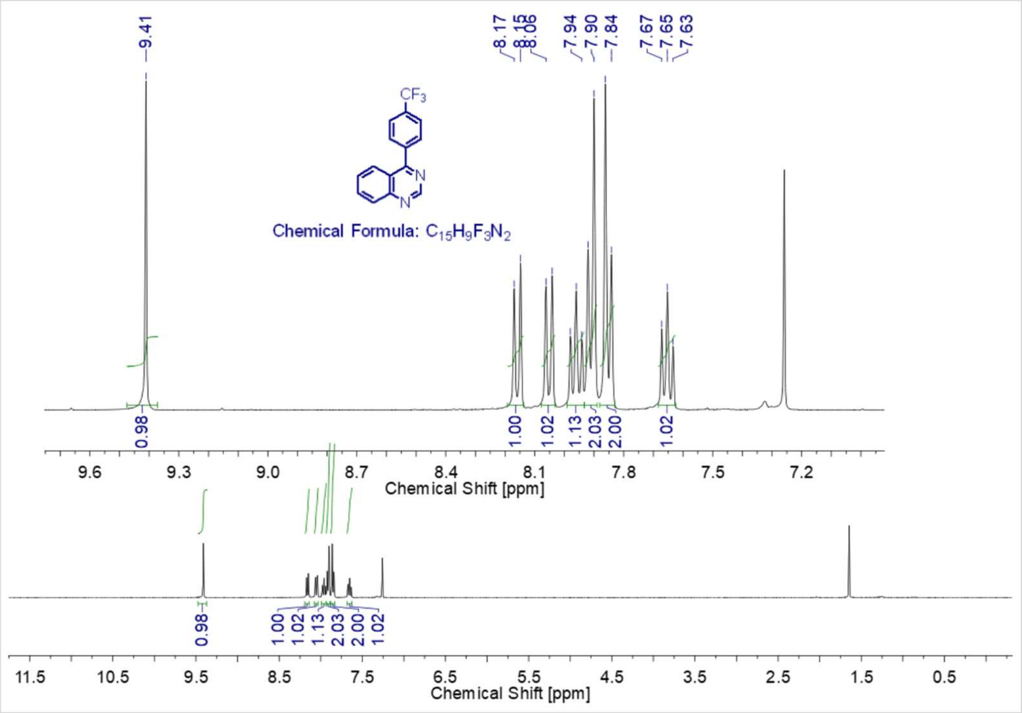


Supplementary Figure 38. $^1$HNMR spectra for **Compound 1**

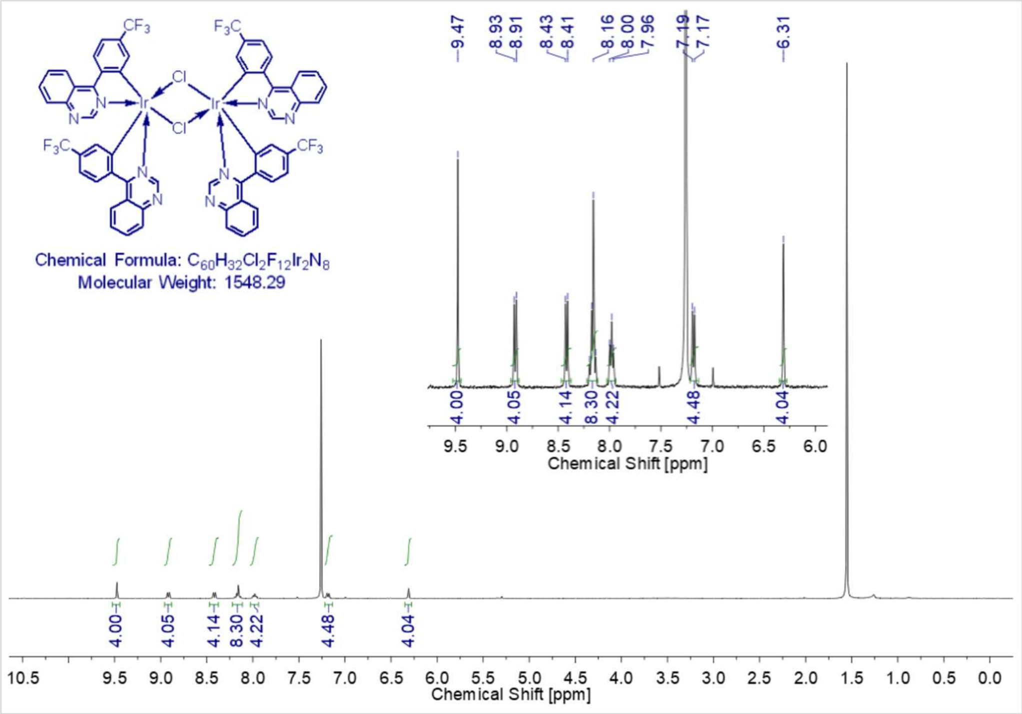


Supplementary Figure 39. $^1$HNMR spectra for **Compound 2**

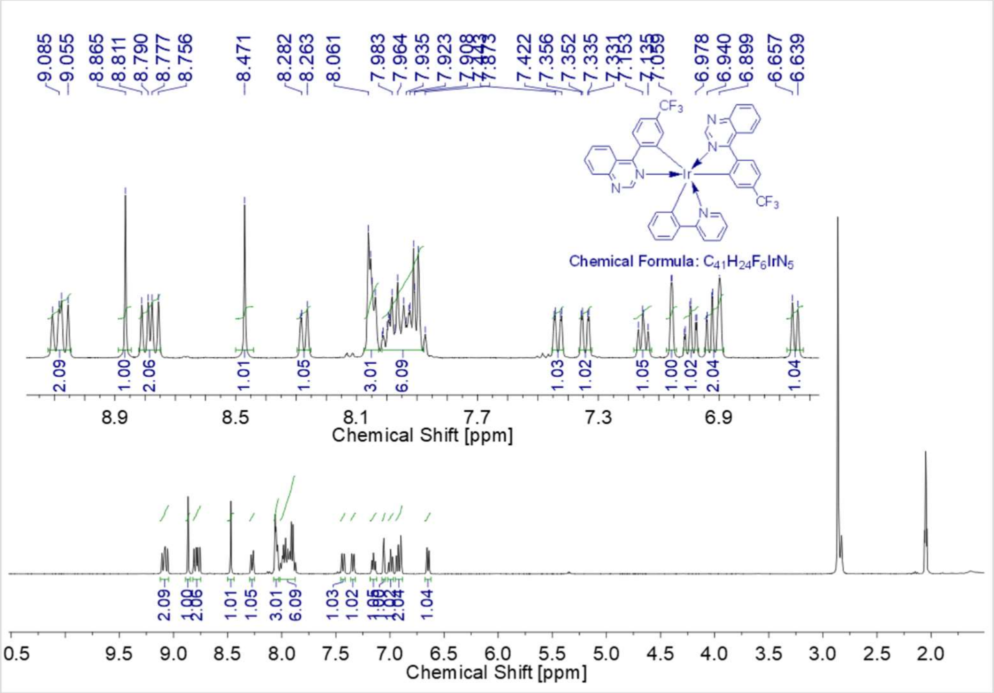


Supplementary Figure 40. $^1$HNMR spectra for **Ir1**

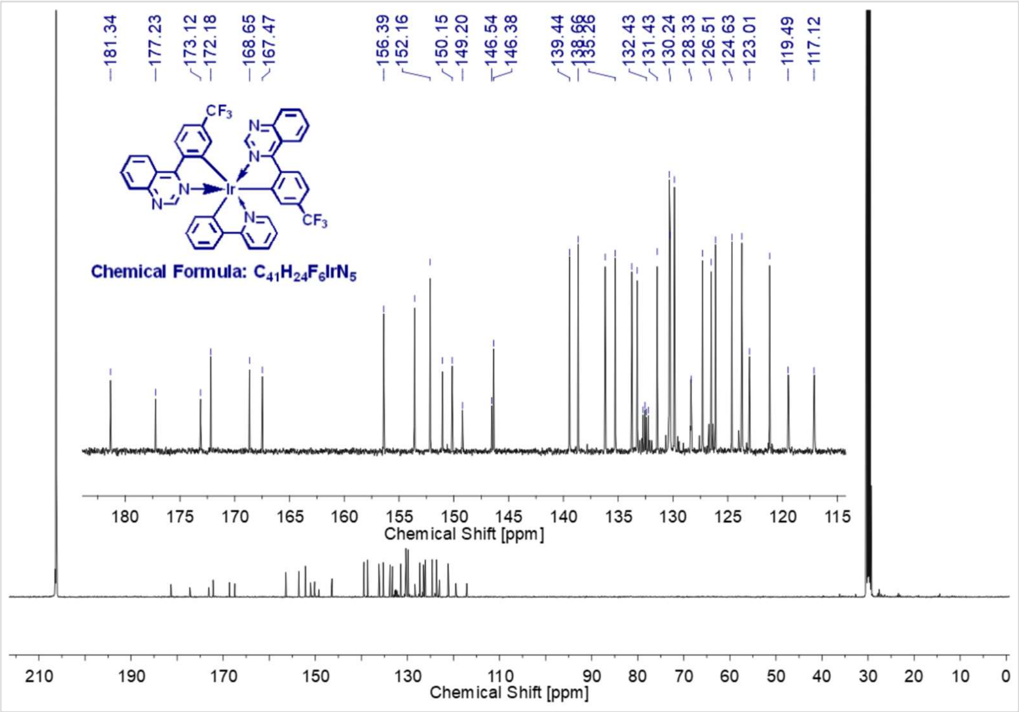


Supplementary Figure 41. $^{13}$CNMR spectra for **Ir1**

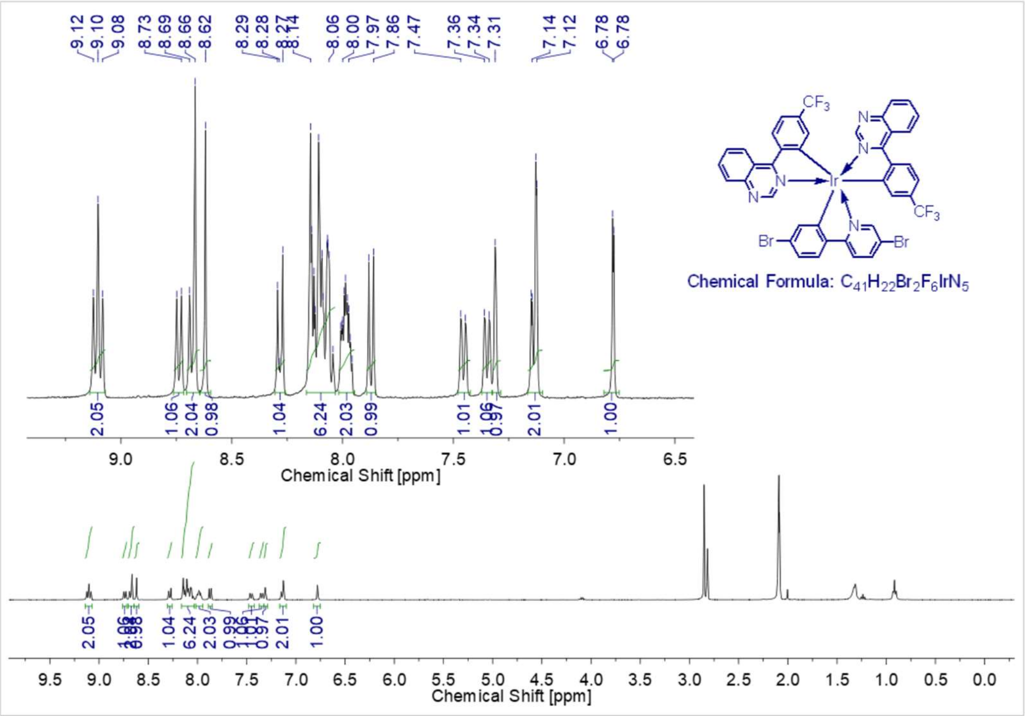


Supplementary Figure 42. $^{1}$HNMR spectra for **Compound 3**

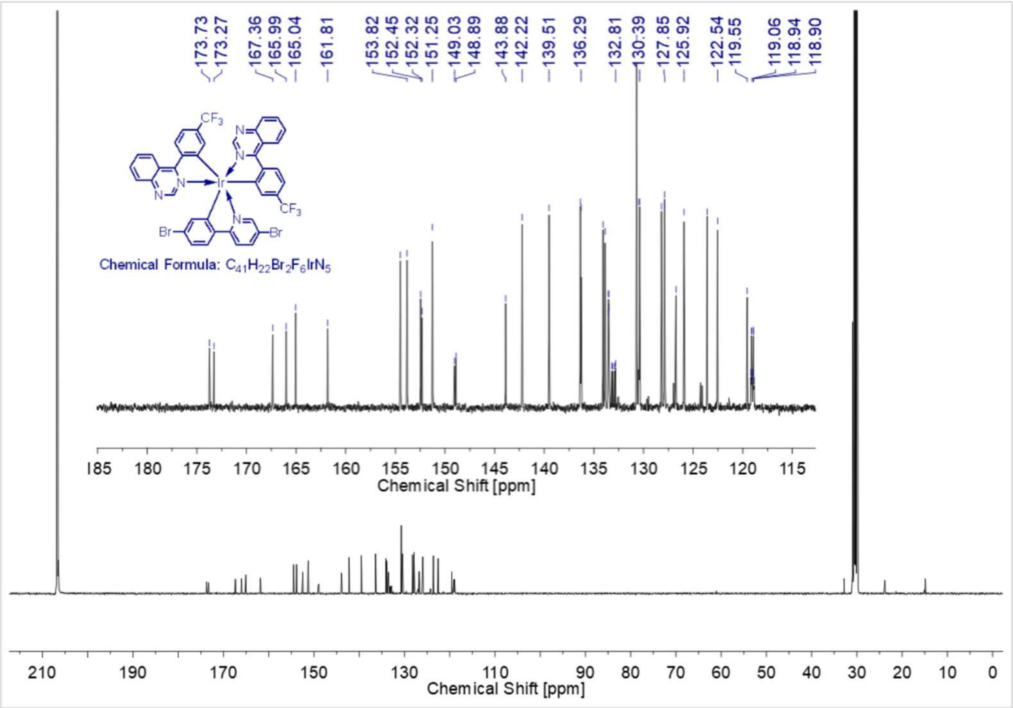


Supplementary Figure 43. $^{13}$CNMR spectra for **Compound 3**

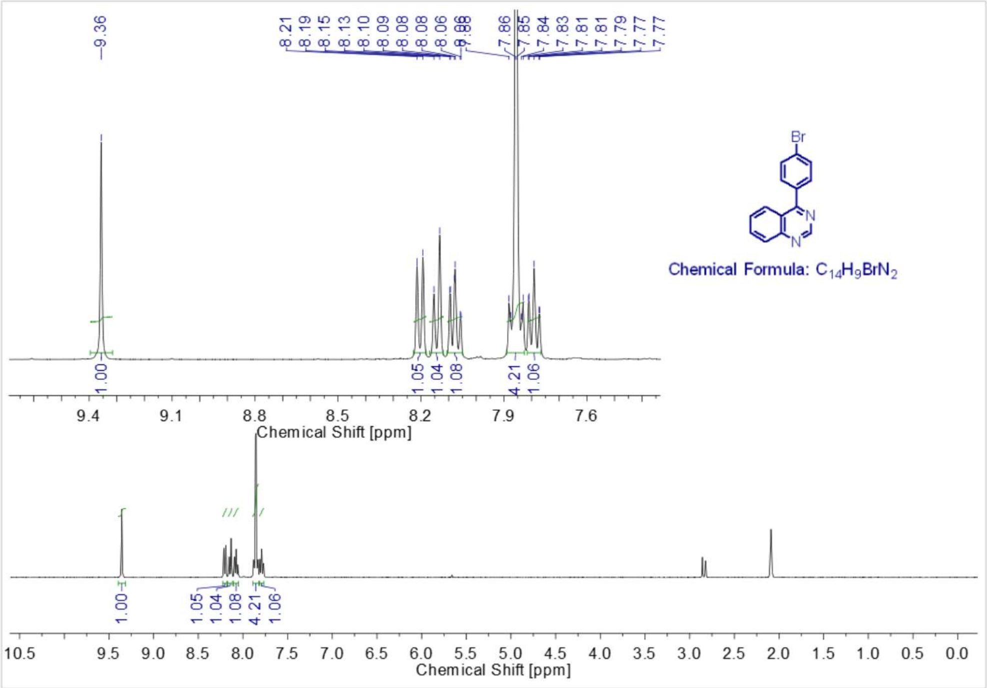


Supplementary Figure 44. $^{1}$HNMR spectra for **Compound 4**

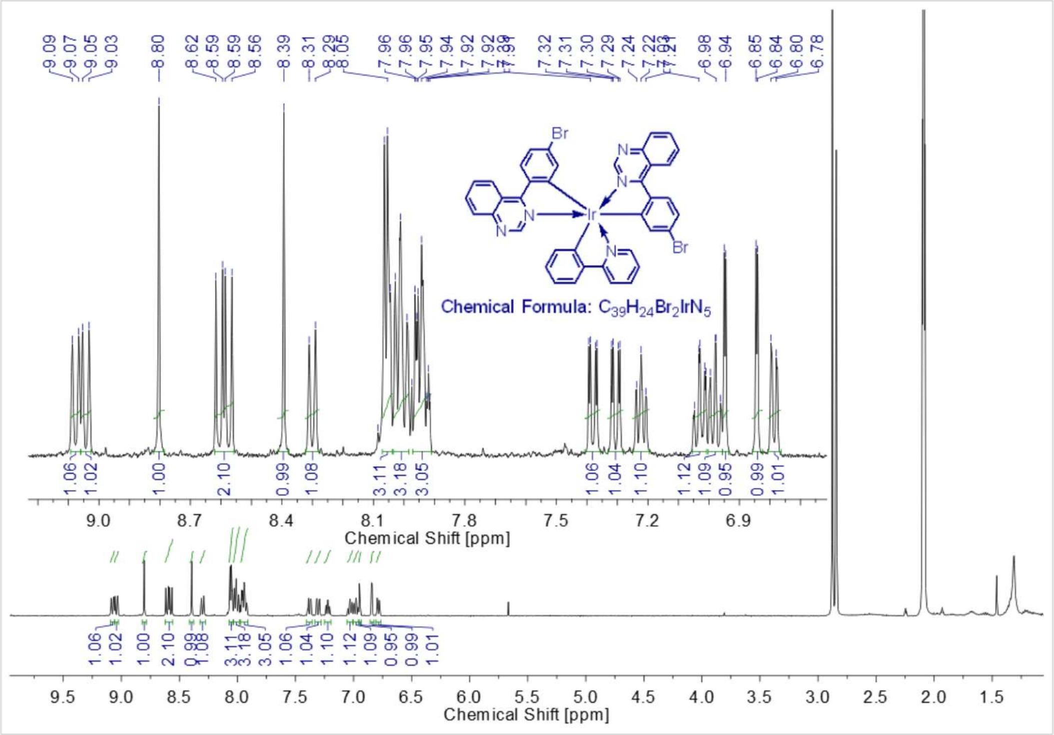


Supplementary Figure 45. $^{1}$HNMR spectra for **Ir2**

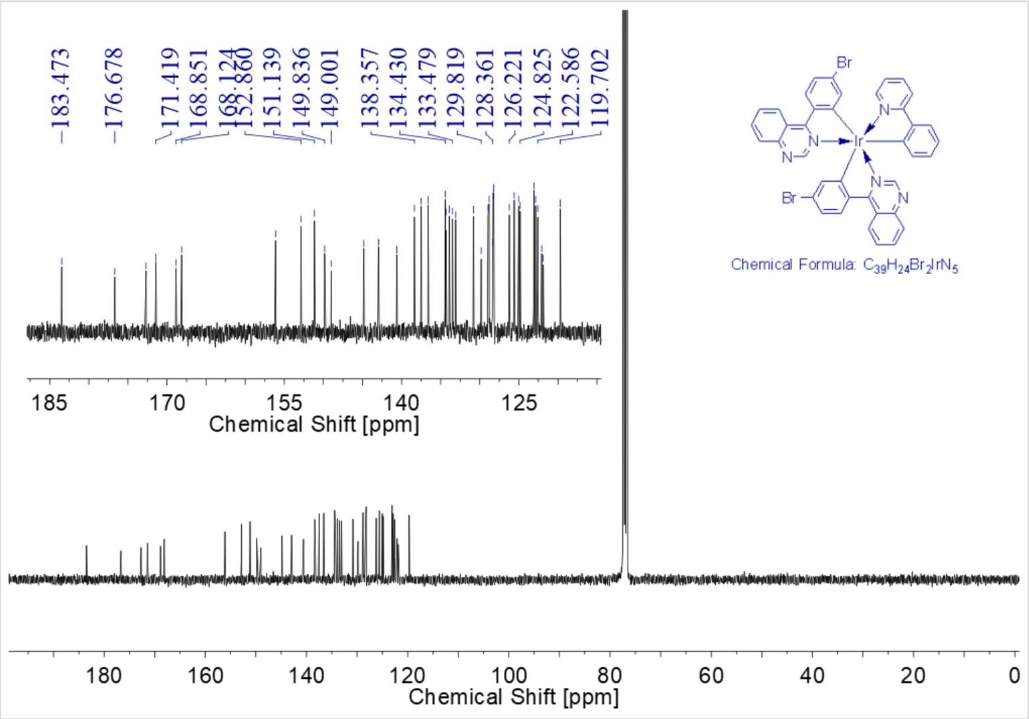


Supplementary Figure 46. $^{13}$CNMR spectra for **Ir2**

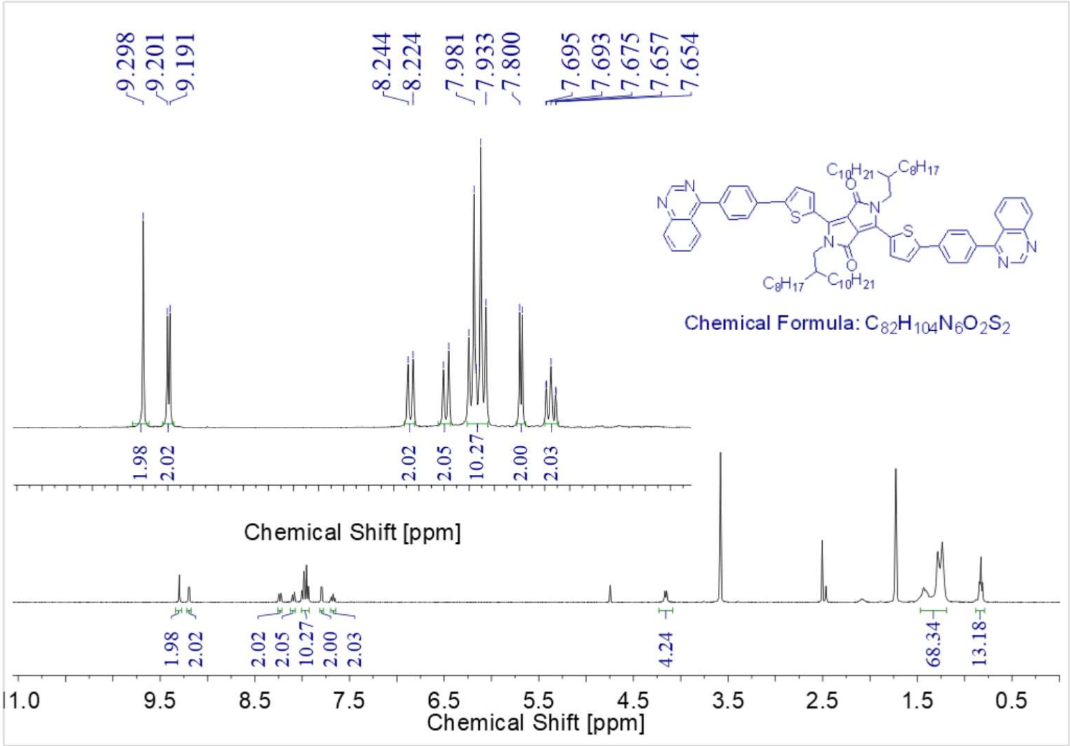


Supplementary Figure 47. $^{1}$HNMR spectra for **QZL-DPP**

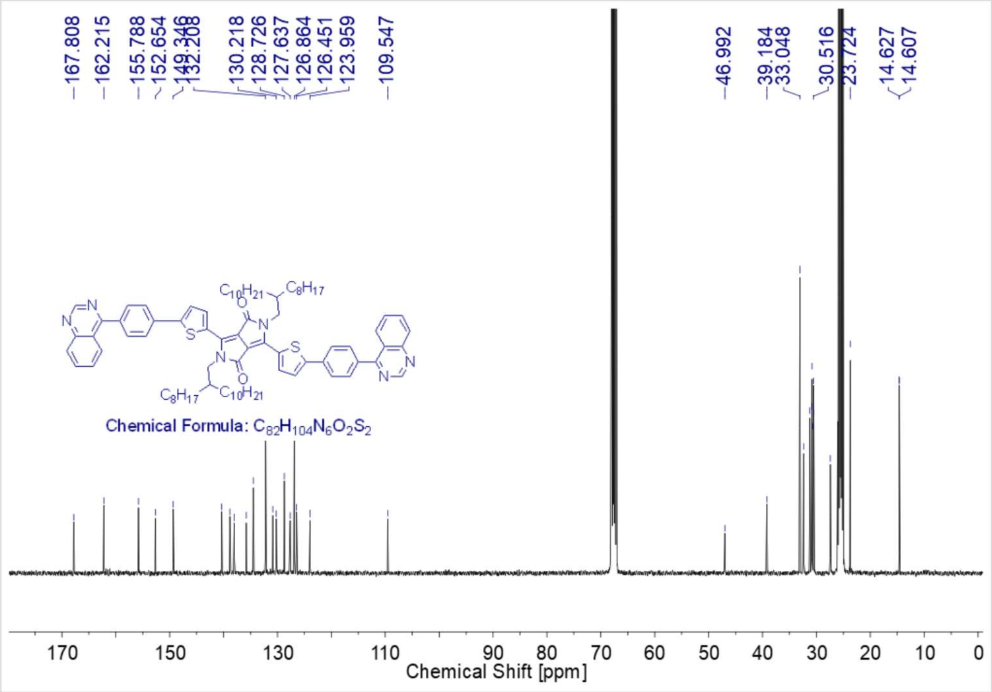


Supplementary Figure 48. $^{13}$CNMR spectra for **QZL-DPP**

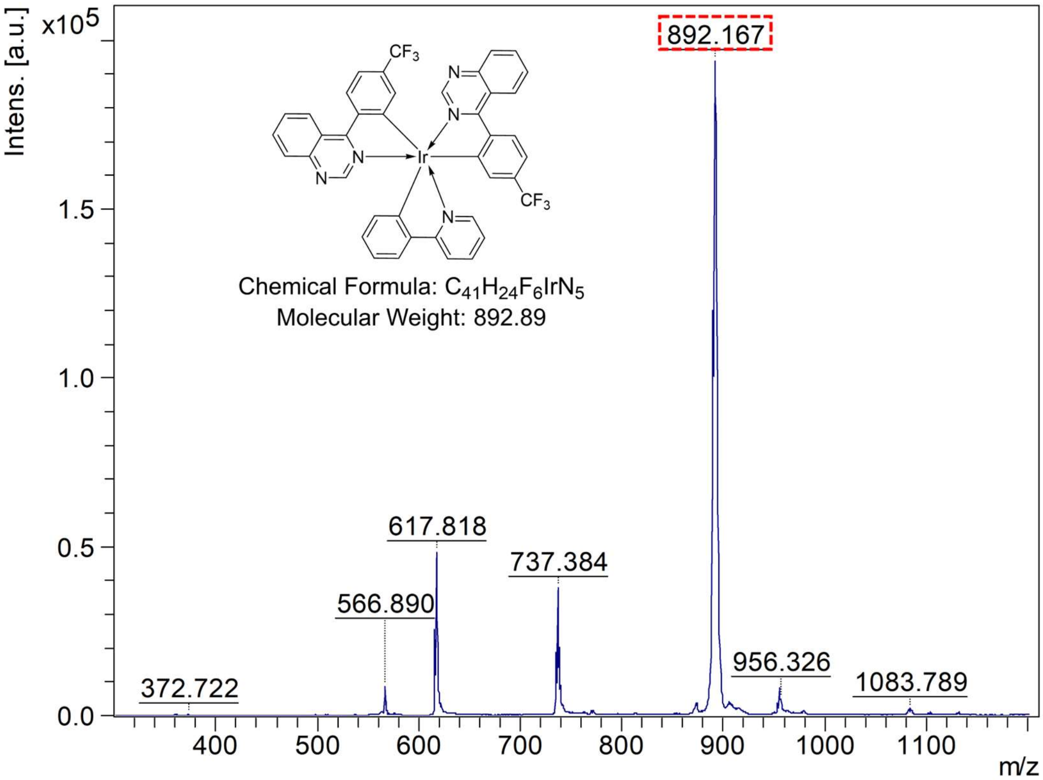


Supplementary Figure 49. Mass spectra for **Ir1**

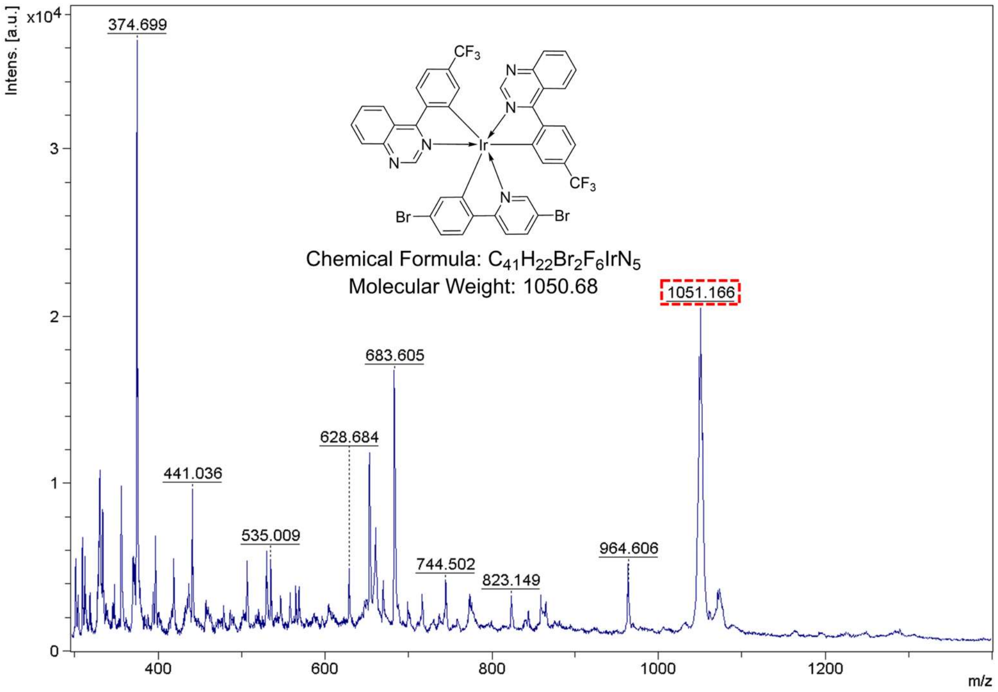


Supplementary Figure 50.· Mass spectra for **Compound 3**

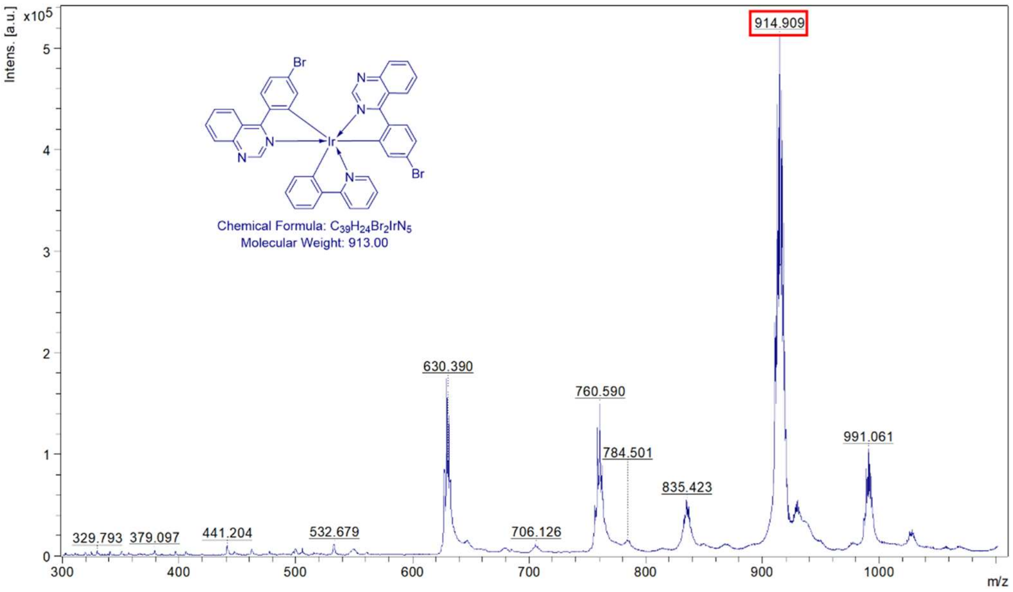


Supplementary Figure 51.· Mass spectra for **Ir2**

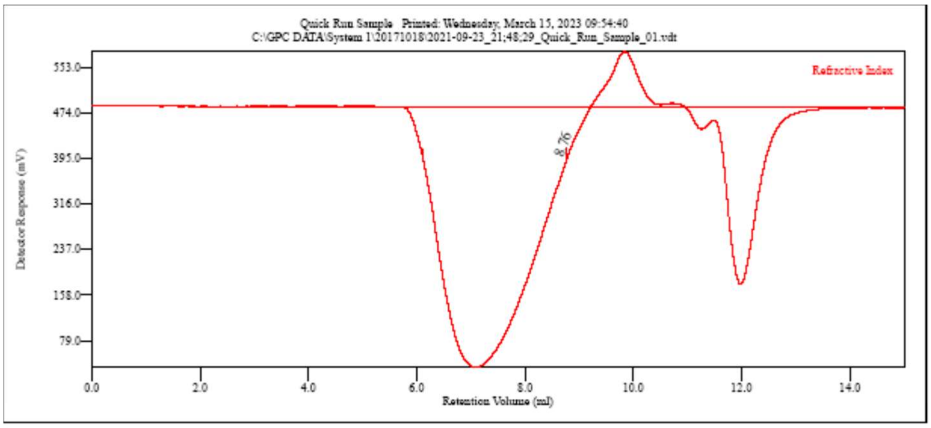


Conventional Calibration - Homopolymers : Results

| | | |
|---|---|---|
| Peak RV - (ml) | | 8.757 |
| Mn - (Daltons) | | 26,698 |
| Mw - (Daltons) | | 68,149 |
| Mz - (Daltons) | | 113,572 |
| Mp - (Daltons) | | 3,808 |
| Mw / Mn | | 2.553 |
| Percent Above Mw: | 0 | 100.000 |
| Percent Below Mw: | 0 | 0.000 |
| Mw 10.0% Low | | 3,820 |
| Mw 10.0% High | | 207,746 |
| Wt Fr (Peak) | | 1.000 |
| RI Area - (mvml) | | -831.66 |
| UV Area - (mvml) | | 0.00 |

| | |
|---|---|
| Annotation | |
| Method File | test-hn-0003.vcm |
| Limits File | |
| Date Acquired | Sep 23, 2021 - 21:48:29 |
| Solvent | THF |
| Acquisition Operator | admin : Administrator |
| Calculation Operator | admin : Administrator |
| Column Set | I-GUAD+IM |
| System | System 1 |
| Flow Rate - (ml/min) | 1.000 |
| Inj Volume - (ul) | 100.0 |
| Volume Increment - (ml) | 0.00333 |
| Detector Temp. - (deg C) | 40.0 |
| Column Temp. - (deg C) | 40.0 |
| OmniSEC Build Number | 467 |

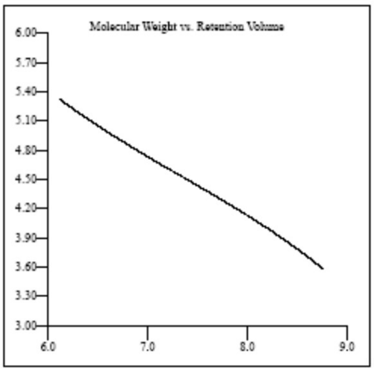


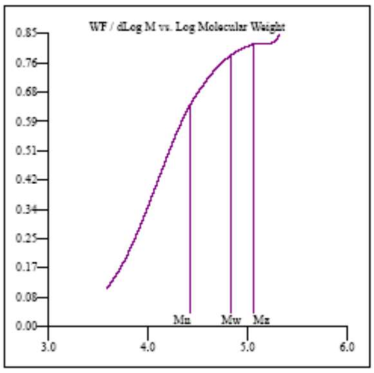


Supplementary Figure 52.· Gel permeation chromatography spectra for **Ir-P1**

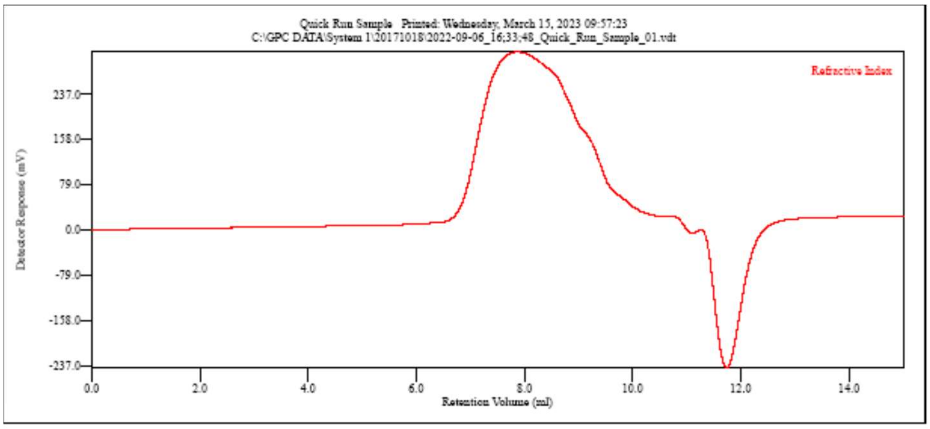


Conventional Calibration - Homopolymers : Results

| | | |
|---|---|---|
| Peak RV - (ml) | | 7.863 |
| Mn - (Daltons) | | 10,920 |
| Mw - (Daltons) | | 19,207 |
| Mz - (Daltons) | | 30,389 |
| Mp - (Daltons) | | 16,343 |
| Mw / Mn | | 1.759 |
| Percent Above Mw: | 0 | 100.000 |
| Percent Below Mw: | 0 | 0.000 |
| Mw 10.0% Low | | 4,086 |
| Mw 10.0% High | | 51,930 |
| Wt Fr (Peak) | | 1.000 |
| RI Area - (mvml) | | 503.35 |
| UV Area - (mvml) | | 0.00 |

| | |
|---|---|
| Annotation | |
| Method File | test-hn-0003.vcm |
| Limits File | |
| Date Acquired | Sep 06, 2022 - 16:33:48 |
| Solvent | THF |
| Acquisition Operator | admin : Administrator |
| Calculation Operator | admin : Administrator |
| Column Set | I-GUAD+IM |
| System | System 1 |
| Flow Rate - (ml/min) | 1.000 |
| Inj Volume - (ul) | 100.0 |
| Volume Increment - (ml) | 0.00333 |
| Detector Temp. - (deg C) | 40.0 |
| Column Temp. - (deg C) | 40.0 |
| OmniSEC Build Number | 467 |

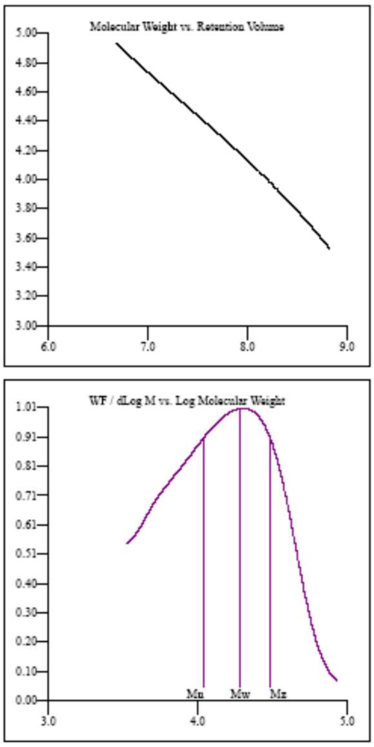


Supplementary Figure 53.· Gel permeation chromatography spectra for **Ir-P2**

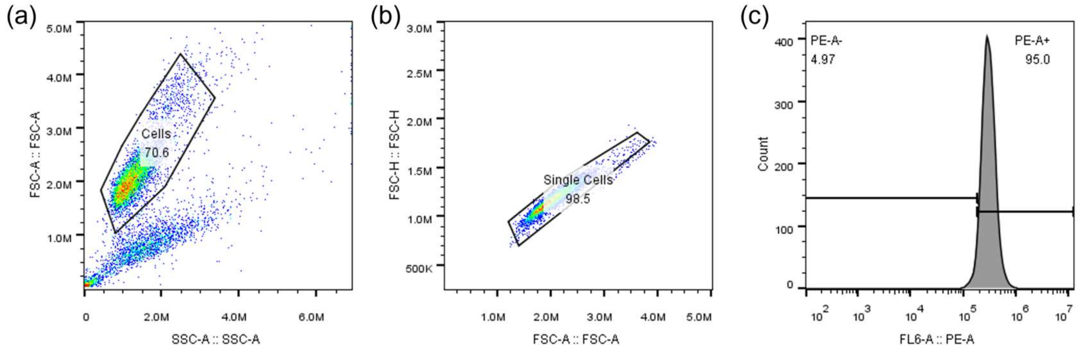


Supplementary Figure 54. Gating strategy for flow cytometry. Step 1, using forward and side scatter gating to extract signals in (a); Step 2, using pulse geometry gating to extract single cells in (b); Step 3, fluorescence analysis of specific channel in (c).

## Supplementary References

1. Zhou T, Hu R, Wang L, Qiu Y, Zhang G, Deng Q, Zhang H, Yin P, Situ B, Zhan C, Qin A, Tang BZ. An AIE-Active Conjugated Polymer with High ROS-Generation Ability and Biocompatibility for Efficient Photodynamic Therapy of Bacterial Infections. *Angewandte Chemie International Edition* **59**, 9952-9956 (2020).
