# Supplementary material for: Metallopolymer strategy to explore hypoxic active narrow-bandgap photosensitizers for effective cancer photodynamic therapy
Source: Nat Commun. 2024 Jan 2;15:170. doi: 10.1038/s41467-023-43890-z (PMC10762066; doi:10.1038/s41467-023-43890-z)
Supplement: Supplementary file 3 — Reporting Summary [file 41467_2023_43890_MOESM3_ESM.pdf]

nature portfolio

Corresponding author(s): Prof. Xuanjun ZHANG

Last updated by author(s): Nov 18, 2023

Reporting Summary

Nature Portfolio wishes to improve the reproducibility of the work that we publish. This form provides structure for consistency and transparency in reporting. For further information on Nature Portfolio policies, see our Editorial Policies and the Editorial Policy Checklist.

Statistics

For all statistical analyses, confirm that the following items are present in the figure legend, table legend, main text, or Methods section.

n/a	Confirmed	
[ ]	[x]	The exact sample size (*n*) for each experimental group/condition, given as a discrete number and unit of measurement
[ ]	[x]	A statement on whether measurements were taken from distinct samples or whether the same sample was measured repeatedly
[ ]	[x]	The statistical test(s) used AND whether they are one- or two-sided<br>*Only common tests should be described solely by name; describe more complex techniques in the Methods section.*
[x]	[ ]	A description of all covariates tested
[x]	[ ]	A description of any assumptions or corrections, such as tests of normality and adjustment for multiple comparisons
[ ]	[x]	A full description of the statistical parameters including central tendency (e.g. means) or other basic estimates (e.g. regression coefficient) AND variation (e.g. standard deviation) or associated estimates of uncertainty (e.g. confidence intervals)
[ ]	[x]	For null hypothesis testing, the test statistic (e.g. *F*, *t*, *r*) with confidence intervals, effect sizes, degrees of freedom and *P* value noted<br>*Give P values as exact values whenever suitable.*
[x]	[ ]	For Bayesian analysis, information on the choice of priors and Markov chain Monte Carlo settings
[x]	[ ]	For hierarchical and complex designs, identification of the appropriate level for tests and full reporting of outcomes
[x]	[ ]	Estimates of effect sizes (e.g. Cohen's *d*, Pearson's *r*), indicating how they were calculated

Our web collection on statistics for biologists contains articles on many of the points above.

Software and code

Policy information about availability of computer code

Data collection	1H and 13C NMR spectra were measured on a Bruker AV-400 MHz NMR spectrometer. Mass spectra were recorded on Bruker Microflex MALDI-TOF system. Uv-Vis absorption spectra were measured on a Shimadzu UV-1800 spectrometer. Photoluminescence spectra were conducted on the Horiba Fluorolog-3 spectrofluorometer. Gel permeation chromatography was run on Malvern Viscotek TDA 305. Particle sizes and surface potential measurements were performed on the Malvern Zetasizer Nano ZS. Confocal laser scanning microscope (CLSM) was performed on Nikon A1R Confocal System. Photoacoustic characterization in solution and imaging in vivo were performed on the Vevo LAZR system (FUJIFILM VisualSonics, Toronto, Canada). The femtosecond transient absorption (TA) spectra of different samples were taken using an Ultrafast System HELIOS TA spectrometer. Western Blot images were captured and analyzed with Image Bio-Rad Lab™ software.
Data analysis	Ploted and Calculated using Microsoft Excel, Origin 2018 and Grapgpad Prism 9. All NMR data processing was carried out using MestReNova. Flow cytometry data processed using FlowJo. UV-Vis and fluorescence spectra were processed by Origin 2018. Statistics calculated in Excel. Cofocal images were processed with NIS Viewer. Photoacoustic results were processed in PowerPoint. Transient absorption spectra data were processed in Origin 2018. Tissue slices were analyzed with SlideViewers. Western Blot images were captured and analyzed with Image Bio-Rad Lab™ software.

For manuscripts utilizing custom algorithms or software that are central to the research but not yet described in published literature, software must be made available to editors and reviewers. We strongly encourage code deposition in a community repository (e.g. GitHub). See the Nature Portfolio guidelines for submitting code & software for further information.

Data

Policy information about availability of data

All manuscripts must include a data availability statement. This statement should provide the following information, where applicable:

- Accession codes, unique identifiers, or web links for publicly available datasets
- A description of any restrictions on data availability
- For clinical datasets or third party data, please ensure that the statement adheres to our policy

The data that supports the findings of this study can be found in the manuscript, and its Supplementary Information, or are available from the corresponding author upon request.

Research involving human participants, their data, or biological material

Policy information about studies with human participants or human data. See also policy information about sex, gender (identity/presentation), and sexual orientation and race, ethnicity and racism.

Reporting on sex and gender	No research involving human participants, their data, or biological material
Reporting on race, ethnicity, or other socially relevant groupings	No research involving human participants, their data, or biological material
Population characteristics	No research involving human participants, their data, or biological material
Recruitment	No research involving human participants, their data, or biological material
Ethics oversight	No research involving human participants, their data, or biological material

Note that full information on the approval of the study protocol must also be provided in the manuscript.

Field-specific reporting

Please select the one below that is the best fit for your research. If you are not sure, read the appropriate sections before making your selection.

☒ Life sciences ☐ Behavioural & social sciences ☐ Ecological, evolutionary & environmental sciences

For a reference copy of the document with all sections, see nature.com/documents/nr-reporting-summary-flat.pdf

Life sciences study design

All studies must disclose on these points even when the disclosure is negative.

Sample size	Sample size for in vitro dark- and photo-cytotoxicity assays triplicates of n = 4. Sample sizes for in vivo tumor cell killing evaluation n = 6, including tumor volume and tumor weight measuring. No statistical methods were used to predetermine sample sizes. All of our experiments followed well-established reported protocols.
Data exclusions	No data was excluded.
Replication	ROS assay in vitro experiments were repeated three times independently with similar results. Western blot analysis was repeated three times independently with similar results. Living/dead cell staining was repeated independently two times with similar results. Cell viability assay was repeated three times independently of n = 4 with similar results. Tumor volume and weight data were acquired from n = 6 mice. Tissue slices (n = 3) were prepared and scanned with similar results.
Randomization	For in vitro experiments, 2 groups of 4 total groups were randomly added with MPdots; further 1 group of 2 groups was randomly irradiated with laser, and 1 group in undosed 2 groups was irradiated with laser. For in vivo experiments, mice were temporally gathered in one cage and randomly distributed into four groups on the 7th day.
Blinding	No blinding was used for in vitro experiments. For in vivo experiments, the investigators were blinded to group allocation during data collection and analysis.

Reporting for specific materials, systems and methods

We require information from authors about some types of materials, experimental systems and methods used in many studies. Here, indicate whether each material, system or method listed is relevant to your study. If you are not sure if a list item applies to your research, read the appropriate section before selecting a response.

Materials & experimental systems

n/a	Involved in the study	
[ ]	[x]	Antibodies
[ ]	[x]	Eukaryotic cell lines
[x]	[ ]	Palaeontology and archaeology
[ ]	[x]	Animals and other organisms
[x]	[ ]	Clinical data
[x]	[ ]	Dual use research of concern
[x]	[ ]	Plants

Methods

n/a	Involved in the study	
[x]	[ ]	ChIP-seq
[ ]	[x]	Flow cytometry
[x]	[ ]	MRI-based neuroimaging

Antibodies

Antibodies used	BcL-2 primary antibody: San Yao Hong Co. Lda (supplier); Lot#B2117; sc-7382; Santa Cruz Biotechnology, Inc; 1:2000 dilution<br>Anti-β-actin: San Yao Hong Co. Lda (supplier); Catalog # 3700, clone 8H10D10, Cell signaling technology; dilution ratio 1:2000<br>Rabbit anti-Mouse IgG Secondary Antibody: San Yao Hong Co. Lda (supplier); Catalog # 61-6520, Invitrogen; 1:1000 dilution
Validation	Bcl-2 is one among many key regulators of apoptosis, which are essential for proper development, tissue homeostasis, and protection against foreign pathogens. https://datasheets.scbt.com/sc-7382.pdf

Eukaryotic cell lines

Policy information about cell lines and Sex and Gender in Research

Cell line source(s)	HeLa cells and 4T1 cells were obtained from the Faculty of Health Science, University of Macau.
Authentication	HeLa cells and 4T1 cells were obtained from the Faculty of Health Science, University of Macau. The parental cell lines were authenticated by the supplier (ATCC).
Mycoplasma contamination	All cell lines were tested negative for mycoplasma contamination.
Commonly misidentified lines (See ICLAC register)	HeLa cell line itself is the contaminating cell line to many misidentified lines, so it is hard to be misidentified. HeLa is known to be an extremely aggressive and fast growing cancer cell line, so it is used in the research.

Animals and other research organisms

Policy information about studies involving animals; ARRIVE guidelines recommended for reporting animal research, and Sex and Gender in Research

Laboratory animals	Female BALB/c mice (5 weeks old) were provided by the animal facility of the University of Macau. All animal procedures were approved by the Institutional Animal Care and Use Committee of the University of Macau (approval number: UMARE-013-2022). Animals were bred and housed with a light cycle of 12:12, ambient temperature at 22 degree Celsius, and relative humidity rang between 40-70%.
Wild animals	The study did not involve wild animals.
Reporting on sex	Female BALB/c mice were used for 4T1 breast tumor model.
Field-collected samples	The study did not involve samples collected from the field.
Ethics oversight	All animal procedures were approved by the Institutional Animal Care and Use Committee of the University of Macau (approval number: UMARE-013-2022).

Note that full information on the approval of the study protocol must also be provided in the manuscript.

Flow Cytometry

Plots

Confirm that:

- [x] The axis labels state the marker and fluorochrome used (e.g. CD4-FITC).
- [x] The axis scales are clearly visible. Include numbers along axes only for bottom left plot of group (a 'group' is an analysis of identical markers).
- [x] All plots are contour plots with outliers or pseudocolor plots.
- [x] A numerical value for number of cells or percentage (with statistics) is provided.

Methodology

Sample preparation	HeLa cells were seeded in 12-well plates at a density of 50,000 cells/mL per well 16 hours in advance and divided into four groups with different treatments. MPdot coated with R8 in deionized water was diluted with DMEM to a final concentration of 20 μg/mL and used to incubate the cells in MPdot groups for 20 hours. All cells were then washed with PBS for three times and incubated with Rhodamine 123 (5μM) in DMEM without FBS for 0.5 hour. The cells were further washed with PBS for three times and the cells in laser groups were irradiated with a 680 nm laser at the power of 40 mW·cm-2 for 5 mins. Then all cells were washed with fresh PBS, digested with trypsin and collected for further detection.
Instrument	The flow cytometry experiments were run on CytoFLEX Flow Cytometer, Beckman.
Software	The flow cytometry data was collected on CytoFLEX Flow Cytometer, Beckman and analyzed on FlowJo X.
Cell population abundance	10000 cells were measured on the CytoFLEX Flow Cytometer system. The signal acquired was extracted based on FSC/SSC gating strategy; and further 95% signals as single cells' signal was extracted using pulse geometry gating strategy.
Gating strategy	Step 1, using forward and side scatter gating to extract signals; step 2, using pulse geometry gating to extract single cells; step 3, fluorescence analysis of specific channel.

☒ Tick this box to confirm that a figure exemplifying the gating strategy is provided in the Supplementary Information.
